# Earth's changing global atmospheric energy cycle in response to climate change

**DOI:** 10.1038/ncomms14367

**Published:** 2017-01-24

**Authors:** Yefeng Pan, Liming Li, Xun Jiang, Gan Li, Wentao Zhang, Xinyue Wang, Andrew P. Ingersoll

**Affiliations:** 1 Department of Physics, University of Houston, 4800 Calhoun Road, Houston, Texas 77204, USA; 2 Department of Earth and Atmospheric Sciences, University of Houston, Houston, Texas 77204, USA; 3 School of Electronic Engineering and automation, Guilin University of Electronic Technology, Guilin, Guangxi 541000; 4 Institute of information Technology, Guilin University of Electronic Technology, Guilin, Guangxi 541000, China; 5 Division of Geological and Planetary Sciences, Caltech, Pasadena, California 91125, USA

## Abstract

The Lorenz energy cycle is widely used to investigate atmospheres and climates on planets. However, the long-term temporal variations of such an energy cycle have not yet been explored. Here we use three independent meteorological data sets from the modern satellite era, to examine the temporal characteristics of the Lorenz energy cycle of Earth's global atmosphere in response to climate change. The total mechanical energy of the global atmosphere basically remains constant with time, but the global-average eddy energies show significant positive trends. The spatial investigations suggest that these positive trends are concentrated in the Southern Hemisphere. Significant positive trends are also found in the conversion, generation and dissipation rates of energies. The positive trends in the dissipation rates of kinetic energies suggest that the efficiency of the global atmosphere as a heat engine increased during the modern satellite era.

## Abstract (web summary)

The long-term Lorenz energy cycle of Earth's global remains poorly explored. Here, the authors use three independent meteorological data sets from the modern satellite era (1979–2013) to examine the temporal characteristics of such a cycle.

Atmospheric energetics describe the roles of different energies in the atmospheric system. Among atmospheric energies, the mechanical energies (that is, the potential energy and the kinetic energy) are mainly related to the atmospheric movement and circulation, which influence weather and climate. Lorenz provided the first modern picture of the mechanical energies and their conversions for the global atmosphere^1^, which is called the Lorenz energy cycle. Such an energy cycle can help diagnose the atmospheric dynamics and general circulation, which is widely used in the studies of the atmospheres of Earth^2^,^3^,^4^,^5^,^6^,^7^,^8^,^9^,^10^,^11^,^12^,^13^,^14^ and other planets^15^,^16^,^17^. Furthermore, the basic idea of atmospheric energies suggested by Lorenz^1^ was extended to other energy concepts^18^,^19^,^20^,^21^,^22^,^23^,^24^ and applied to the studies of hydrological cycle, climate change and alternative energy sources^25^,^26^,^27^,^28^,^29^,^30^,^31^,^32^.

The mean states of the Lorenz energy cycle of Earth's global atmosphere were investigated in previous studies^5^,^9^,^12^,^33^,^34^. In addition, the temporal variability of the Lorenz energy cycle was explored with a 5-year (1958–1963) data set^7^ and a 10-year (1963–1973) data set^33^. However, studies of the long-term temporal characteristics of the global atmospheric energy cycle are lacking. Here we examine the linear trends of the Lorenz energy cycle of the global atmosphere in the modern satellite era (1979–2013) with two satellite-based meteorological data sets (see Methods): the newest reanalysis from the National Centers of Environmental Prediction and the Department of Energy Reanalysis II (NCEP-DOE R2)^35^,^36^,^37^ and the European Centre for Medium-Range Weather Forecasts Re-Analysis Interim (ERA-Interim)^38^,^39^,^40^. In addition, the National Aeronautics and Space Administration (NASA) Modern-Era Retrospective Analysis for Research and Applications Version 2 (MERRA-2)^41^,^42^, which has the data beginning in 1980, is used to validate the results from the two independent data sets (that is, NCEP-DOE R2 and ERA-Interim).

The NCEP-DOE R2 data set was initialized by the NCEP and the National Center for Atmospheric Research reanalysis^35^. With significant improvements from the NCEP and National Center for Atmospheric Research reanalysis, the NCEP-DOE R2 is becoming a standard reanalysis data set in the community of atmospheric science and climate change in the United States^37^. The ERA-Interim is the latest version of the standard meteorological data set from the European Centre for Medium-Range Weather Forecasts, which is based on its previous reanalysis of the global atmosphere and surface conditions^38^. The NASA MERRA-2 (ref. 42) was introduced to replace the original MERRA^41^ data set because of advances made in the assimilation system. The three reanalysis data sets are the best publicly available data sets for the long-term global meteorological conditions, which include the necessary variables (for example, three-dimensional wind field, temperature field and geo-potential height field) with a daily time step to compute the Lorenz energy cycle of the global atmosphere. The three data sets are obtained by assimilating satellite data into state-of-the-art analysis/forecast models, which makes them physically consistent. In addition, the three satellite-based data sets, covering a much longer time period than the 5-year and 10-year global data sets used in the previous studies^7^,^33^, provide a great opportunity to study the temporal characteristics of the global atmospheric energy cycle. Our following discussions are mainly based on NCEP-DOE R2 and ERA-Interim data sets in the modern satellite era (1979–2013). The NASA MERRA-2 data set, which has a different time period (1980–2013), is used to validate the results from the first two data sets NCEP-DOE R2 and ERA-Interim (Supplementary Figs 10–13).

The formulation of the energy cycle in a mixed space–time domain, which was developed by Oort^19^, and Peixoto and Oort^5^ from Lorenz's theoretical frame^1^, is widely used in the current analyses of the energy components of atmospheres^5^,^6^,^7^,^8^,^9^,^10^,^11^,^12^,^13^,^14^,^15^,^16^,^17^. Here we follow the classical formulation^5^ to calculate the following energies in the energy cycle: the mean available potential energy *P*_M_, the eddy available potential energy *P*_E_, the mean kinetic energy *K*_M_ and the eddy kinetic energy *K*_E_. We also compute the conversion rates among different energies:*C*(*P*_M_, *P*_E_), *C*(*P*_E_, *K*_E_), *C*(*K*_E_, *K*_M_) and *C*(*P*_M_, *K*_M_). Finally, the generation rates of the mean and eddy available potential energies (*G*(*P*_M_) and *G*(*P*_E_)) and the dissipation rates of the mean and eddy kinetic energies (*D*(*K*_M_) and *D*(*K*_E_)) are evaluated from the corresponding conversion rates and the time derivatives of energies. More details of computing the energy components of the Lorenz energy cycle of the global atmosphere are provided in the section on Methods. Our analyses suggest that most energy components in the Lorenz energy cycle have positive trends. As a result, the efficiency of Earth's global atmosphere as a heat engine increased during the past 35 years (1979–2013).

## Results

### Linear trends in the mechanical energies

The latest versions of the two reanalysis data sets (that is,, NCEP-DOE R2 and ERA-Interim) are combined with the theoretical frame to investigate the Lorenz energy cycle of the global atmosphere and its spatio-temporal variabilities. First, we average the energy cycle over the 35-year time period to update the time-mean state of the Lorenz energy cycle (Supplementary Fig. 1), which is basically consistent with our previous results^9^. The time-mean state is further used as a reference for the discussions of the temporal variations of the Lorenz energy cycle in the global atmosphere.

**Figure 1:**
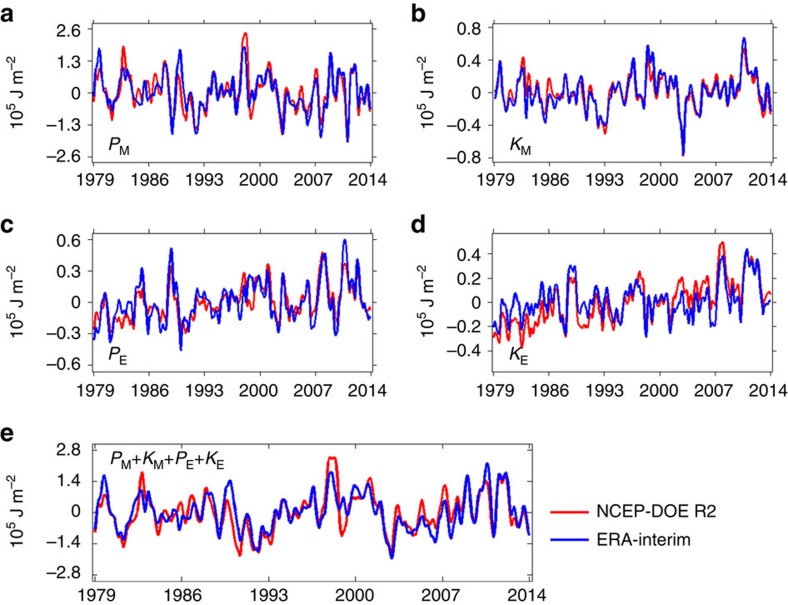
Time series of the global-average atmospheric energies. (**a**) The mean available potential energy *P*_M_. (**b**) The mean kinetic energy *K*_M_. (**c**) The eddy available potential energy *P*_E_. (**d**) The eddy kinetic energy *K*_E_. (**e**) The total mechanical energy (that is, *P*_M_+*K*_M_+*P*_E_+*K*_E_).

Figure 1 displays the time series of the monthly evaluation of the global-average mechanical energies in Earth's atmosphere between 1979 and 2013. The time series of mechanical energies of the global atmosphere display not only the long-term linear trends but also some inter-annual variabilities (for example, El Niño–Southern Oscillation signals). The inter-annual variabilities of the Lorenz energy cycle were discussed in our previous studiy^10^, so we focus on the linear trends of the energy components in this study. The linear trends of these energies are computed using the least-square method^43^ and the corresponding confidence levels (that is, the probabilities of linear trends with a non-zero slope) are estimated by the Student's *t*-statistics (see Methods). The computed linear trends and the corresponding confidence levels for the time series shown in Fig. 1 are summarized in Table 1. As shown in Table 1, the mean available potential energy *P*_M_ and the mean kinetic energy *K*_M_ do not reveal any significant trends. The eddy available potential energy *P*_E_ displays positive linear trends of 755.5±391.5 and 571.6±433.2 J m^−2^ per year for NCEP-DOE R2 and ERA-Interim, respectively. The confidence levels corresponding to the positive trends in *P*_E_ are 96.4% and 90.3% for NCEP-DOE R2 and ERA-Interim, respectively. Based on the time-mean state of the Lorenz energy cycle of the global atmosphere (Supplementary Fig. 1), the eddy available potential energy increased 5.2±2.7% (NCEP-DOE R2) and 3.0±2.3% (ERA-Interim) over the past 35 years (Supplementary Table 1). The eddy kinetic energy *K*_E_ also shows positive linear trends of 1259.5±480.0 J m^−2^ per year (confidence level 99.2%) for NCEP-DOE R2 and 362.1±304.5 J m^−2^ per year (confidence level 87.8%) for ERA-Interim. As shown in Supplementary Table 1, the eddy kinetic energy increased 7.0±2.8% and 2.0±1.7% over the past 35 years for NCEP-DOE R2 and ERA-Interim, respectively. The mean available potential energy *P*_M_, revealing no statistically significant trends, is dominant among the mechanical energies. Therefore, the total mechanic energy (*P*_M_+*K*_M_+*P*_E_+*K*_E_) does not have any significant trend (Fig. 1e and Table 1). The total available potential energy (*P*_M_+*P*_E_) does not have any significant trend (Supplementary Fig. 2) either. On the other hand, the total kinetic energy (*K*_M_+*K*_E_) increased during the past 35 years (Supplementary Fig. 2) due to the positive trend in the eddy kinetic energy (Fig. 1).

The linear trends in the global-average energies are further investigated by exploring their spatial distribution (Supplementary Figs 3–6). For the mean energies (*P*_M_ and *K*_M_), the distribution of trends in the latitude-pressure cross-section (Supplementary Fig. 3) displays some patterns of positive and negative trends at different regions. However, the coverage of significant trends (that is, confidence level>90%) is very small (Supplementary Fig. 3E–H). The distribution of the linear trends of the eddy energies (*P*_E_ and *K*_E_) in the latitude-pressure cross-section (Supplementary Fig. 4) suggests that the coverage of the significant trends of *P*_E_ and *K*_E_ is much larger in the Southern Hemisphere (SH) than in the Northern Hemisphere (NH). The significant positive trends of *P*_E_ and *K*_E_ in the SH are mainly concentrated in the upper troposphere and stratosphere, which are also the locations of the maxima of the climatological *P*_E_ and *K*_E_. The maxima of the climatological *P*_E_ and *K*_E_ are mainly influenced by the jet stream and the storm track^5^,^9^,^33^, so the temporal variations in the jet stream and the storm track could contribute to these positive trends of *P*_E_ and *K*_E_ in the SH.

As the eddy energies (*P*_E_ and *K*_E_) have three-dimensional structures, we also explore their linear trends in the longitude-latitude cross-section (that is, global map). The global maps of the linear trends of the eddy available potential energy *P*_E_ (Supplementary Fig. 5) suggest that both NCEP-DOE R2 (Supplementary Fig. 5A) and the ERA-Interim (Supplementary Fig. 5B) show the strong positive trends of *P*_E_ over the Asian continent in the NH. The confidence levels (Supplementary Fig. 5C,D) indicate that the strong positive trends over the Asian continent are statistically significant (that is, confidence level>90%). The positive trends of *P*_E_ in Central Asia, especially in West-central Mongolia, are associated with the increasing droughts in these areas^44^, because the increasing droughts can magnify the temperature perturbation and hence cause the increase in *P*_E_. The two data sets (NCEP-DOE R2 and ERA-Interim) also show the relatively weak positive trends over the eastern Pacific Oceans. Such positive trends are related to the intensifying tropical cyclones with global warming^45^,^46^,^47^,^48^,^49^,^50^. In the SH, the eddy potential energy *P*_E_ shows significant positive trends around the latitude band of 60°S, which is the storm track in the SH and near the boundary of Antarctica.

The global maps of the linear trends of the eddy kinetic energy *K*_E_ (Supplementary Fig. 6) show that there are no significant trends of *K*_E_ in the NH, except for the positive trends over the central Pacific Ocean, in which the spatial coverage of the positive trends is larger in ERA-Interim (Supplementary Fig. 6B) than in NCEP-DOE R2 (Supplementary Fig. 6A). The positive trends of *K*_E_ over the central Pacific Ocean, which have qualitative consistency between NCEP-DOE R2 and ERA-Interim, are statistically significant (Supplementary Fig. 6C,D). The positive trends are related to the increased tropical hurricane activities associated with the global warming^44^,^45^,^46^,^47^,^48^,^49^. In the SH, the positive trends are shown in the middle and high latitudes, which include the storm track and the boundary of Antarctica. The storm track and the boundary of Antarctica are the locations of the maxima of the climatological *K*_E_^5^,^9^,^33^. Therefore, the positive trends of *K*_E_ in the middle and high latitudes of the SH are related to the temporal variations of the climatological *K*_E_, which are further associated with the increasing storm activities in the SH storm track areas^51^. In particular, the maxima of the positive trends of *K*_E_ around 250°E and 55°S are roughly consistent with the strongest centre of positive trends in the mean radius and depth of cyclones over the Southern Ocean^52^. The positive trends of *K*_E_ in the middle and high latitudes of the SH have larger spatial coverage and stronger magnitude in NCEP-DOE R2 than in ERA-Interim even though they are qualitatively consistent. The quantitative discrepancy in the SH between ERA-Interim and NCEP-DOE R2 sets arises because there were fewer observations in the SH than in the NH and different data-assimilation techniques were used in the two data sets^37^,^39^,^40^.

### Linear trends in the conversion rates

**Figure 2:**
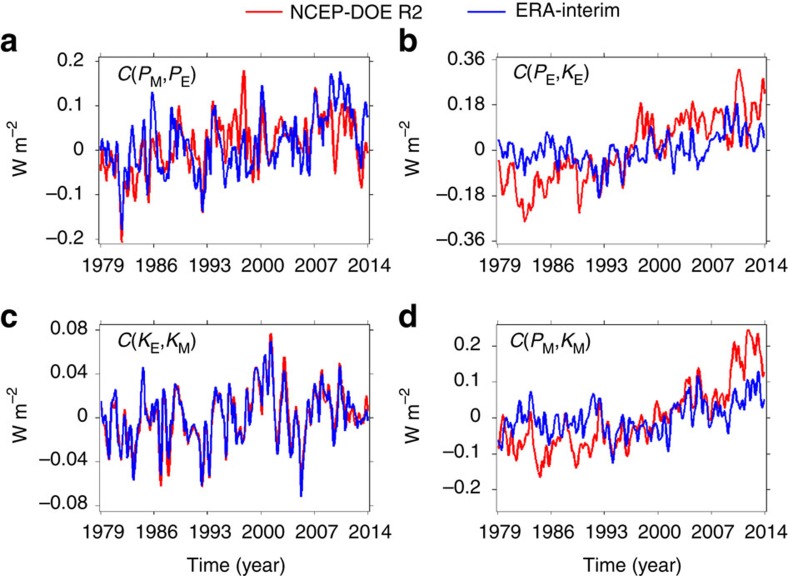
Time series of the global-average conversion rates. (**a**) The conversion rate between the mean available potential energy and the eddy available potential energy *C*(*P*_M_, *P*_E_). (**b**) The conversion rate between the eddy available potential energy and the eddy kinetic energy *C*(*P*_E_, *K*_E_). (**c**) The conversion rate between the eddy kinetic energy and the mean kinetic energy *C*(*K*_E_, *K*_M_). (**d**) The conversion rate between the mean available potential energy and mean kinetic energy *C*(*P*_M_, *K*_M_).

The global-average conversion rates among different energies are displayed in Fig. 2, which suggests that all conversion rates are increasing with time. Table 1 demonstrates that all positive trends in the global-average conversion rates have confidence levels >90%, except for the positive trends of *C*(*K*_E_, *K*_M_) from ERA-Interim (confidence level ∼86.5%). The time-mean state (Supplementary Table 1) further suggests that the conversion rates increased from a few percent to a few tens percent over the past 35 years except for *C*(*K*_E_, *K*_M_) from NCEP-DOE R2 with a varied percentage 122.7±85.2%.

The linear trends of the conversion rates and the corresponding confidence levels are also calculated in the latitude-pressure cross-sections (Supplementary Figs 7 and 8). From Supplementary Fig. 7, we find that the linear trends of the conversion rate *C*(*P*_M_, *P*_E_) have two positive maxima around 70°N and 60°S in the stratosphere, but the two positive maxima are not statistically significant. In the middle troposphere (300–700 mbar), there are positive linear trends of *C*(*P*_M_, *P*_E_) in the middle latitudes of the SH (30–75°S). These positive trends are statistically significant with the spatial coverage larger in NCEP-DOE R2 than in ERA-Interim. These maxima of positive trends have roughly the same locations as the maxima of the climatological *C*(*P*_M_, *P*_E_), which are associated with the middle-latitude cyclones and anticyclones^5^,^9^,^33^. Therefore, the temporal variations of large-scale weather systems in the middle latitudes contribute to the positive trends of *C*(*P*_M_, *P*_E_). In Supplementary Fig. 7, we also shows that the conversion rate *C*(*P*_E_, *K*_E_) has significant positive trends mainly in the SH, which are concentrated around middle latitudes (30–60° N/S) in the stratosphere (10–100 mbar). There are also positive trends of *C*(*P*_E_, *K*_E_) in the upper troposphere (∼300 mbar) around 30° in the two hemispheres. The positive trends of *C*(*P*_E_, *K*_E_) in both troposphere and stratosphere are found around the maxima of the climatological *P*_E_, which are related to jet streams in the troposphere and stratosphere^5^,^9^,^33^. Therefore, the positive trends of *C*(*P*_E_, *K*_E_) are related to the perturbations of the jet streams.

As shown in Supplementary Fig. 8, the positive linear trends of *C*(*K*_E_, *K*_M_) are concentrated in the tropical region (15–30° N/S) of the upper troposphere (100–300 mbar) and the middle latitudes (30–60° N/S) of the upper stratosphere (10–30 mbar). The maxima of the positive trends in *C*(*K*_E_, *K*_M_) shift to the equator relative to the maxima of the climatological *K*_M_, which implies that the linear trends of *C*(*K*_E_, *K*_M_) are associated with the temporal variations of the jet streams in the troposphere and stratosphere. In addition to *C*(*K*_E_, *K*_M_), the spatial patterns of the linear trends of *C*(*P*_M_, *K*_M_) from the two data sets (NCEP-DOE R2 and ERA-Interim) display strong maxima in the middle and high latitudes (50–80° N/S) of the stratosphere. Such maxima are weaker in ERA-Interim than in NCEP-DOE R2. The relatively weaker maxima in ERA-Interim are not statistically significant (Supplementary Fig. 8H). In the troposphere, there are maxima of the positive linear trends of *C*(*P*_M_, *K*_M_) in the tropical region (15–30° N/S) in the upper troposphere (100–300 mbar). Such maxima have the same locations as these of the maxima of the climatological *C*(*P*_M_, *K*_M_), which are associated with the Hadley cell (*C*(*P*_M_, *K*_M_)>0)^5^,^9^,^33^. Therefore, the positive trends of *C*(*P*_M_, *K*_M_) are due to the expansion of the Hadley Cell in the past 35 years, which was revealed in some previous studies^53^,^54^,^55^. In the middle latitudes (30–60° N/S), the linear trends of *C*(*P*_M_, *K*_M_) are relatively complicated in the troposphere. In addition, a large discrepancy between the two data sets (NCEP-DOE R2 and ERA-Interim) is displayed in such a region. The conversion rate *C*(*P*_M_, *K*_M_) in the middle-latitude region is mainly affected by the indirect Ferrel Cell (*C*(*P*_M_, *K*_M_)<0)^5^,^9^,^33^, which is driven by eddies. Therefore, the complicated temporal variations of *C*(*P*_M_, *K*_M_)in this region are related to the complicated eddy activities in the middle latitudes^56^,^57^,^58^.

The global-average time series (Fig. 2) and the spatial distribution (Supplementary Figs 7 and 8) of the conversion rates both suggest that *C*(*P*_E_, *K*_E_) and *C*(*P*_M_, *K*_M_) have relatively large discrepancy between the two data sets (NCEP-DOE R2 and ERA-Interim, whereas *C*(*P*_E_, *K*_E_) and *C*(*K*_E_, *K*_M_) are basically consistent between the two data sets. The large discrepancy in the conversion rates *C*(*P*_E_, *K*_E_) and *C*(*P*_M_, *K*_M_) between the two data sets is probably due to different observational sources and data-assimilation techniques used in the two data sets, as what we discussed above. The investigations of spatial distribution (Supplementary Figs 7 and 8) further suggest that the large discrepancy in *C*(*P*_E_, *K*_E_) and *C*(*P*_M_, *K*_M_) between the two data sets is mainly concentrated near surface of the SH. The paucity of observations over the Southern Oceans, which results in the large uncertainties in validating and assimilating reanalysis data sets, contributes to the discrepancy near surface of the SH between the two data sets.

### Linear trends of generation and dissipation rates

**Figure 3:**
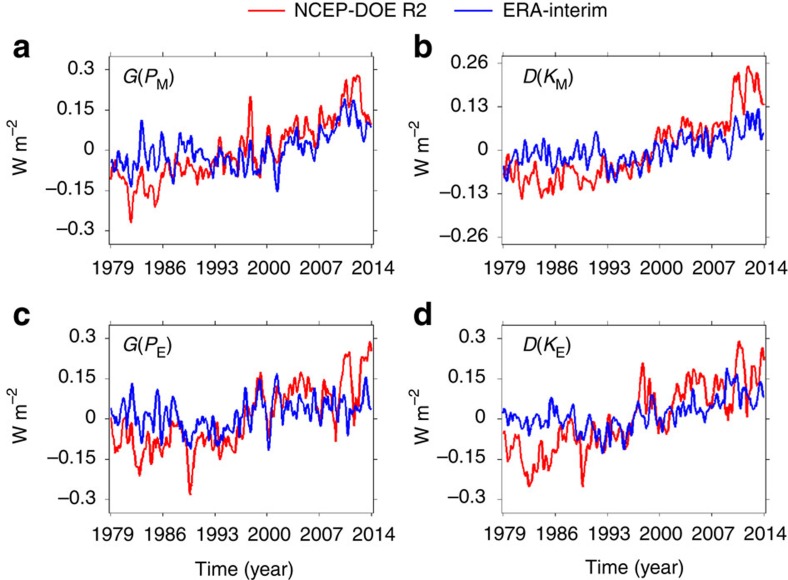
Time series of the global-average generation and dissipation rates. (**a**) The generation rate of the mean available potential energy *G*(*P*_M_). (**b**) The dissipation rate of the mean kinetic energy *D*(*K*_M_). (**c**) The generation rate of the eddy available potential energy *G*(*P*_E_). (**d**) The dissipation rate of the eddy kinetic energy *D*(*K*_E_).

Based on the global-average time differentials of energies (Supplementary Fig. 9) and the conversion rates (Fig. 2), the time series of generation and dissipation rates are generated (see Methods). Figure 3 displays the global-average generation and dissipation rates, which suggests that all generation/dissipation rates are increasing with time. Table 1 further suggests that all positive trends have confidence levels >90% except for the positive trends of *G*(*P*_E_) (confidence level∼81.3%) and *D*(*K*_M_) (confidence level∼87.2%) from ERA-Interim. The positive trends in the generation and dissipation rates are mainly due to the increasing conversion rates, because the time differentials of energies (Supplementary Fig. 9) are at least one order of magnitude smaller than the conversion rates (Fig. 2), so the time differentials of energies do not play significant roles in determining the generation and dissipation rates (see Methods).

The increased dissipation of kinetic energies suggests that the efficiency of the global atmosphere increased if we consider it as a heat engine^34^. There are different ways to define the efficiency of the heat engine of the global atmosphere^15^,^16^,^34^. Here we use a simple definition suggested by Peixoto an Oort^34^, in which the efficiency of the atmosphere is defined as the ratio of the dissipation of kinetic energy and the incoming solar radiation. The incoming solar radiation^34^ is∼238 W m^−2^. As shown in Supplementary Table 1, the time-mean total dissipation rates of kinetic energies (*D*(*K*_M_)+*D*(*K*_E_)) are 2.69±0.20 and 2.02±0.16 W m^−2^ for NCEP-DOE R2 and ERA-Interim, respectively. In Supplementary Fig. 13, we show the time series of *D*(*K*_M_)+*D*(*K*_E_), which are based on the time series of *D*(*K*_M_)and *D*(*K*_E_) (Fig. 3). The linear trends of *D*(*K*_M_)+*D*(*K*_E_) are 0.018±0.007 W m^−2^ per year (confidence level 96.4%) and 0.005±0.002 W m^−2^ per year (confidence level 95.0%) for NCEP-DOE R2 and ERA-Interim, respectively. Integrating the linear trends over the 35-year time period, we have that *D*(*K*_M_)+*D*(*K*_E_)increased by 0.63±0.25 W m^−2^ for NCEP-DOE R2 and 0.18±0.07 W m^−2^ for ERA-Interim during the modern satellite era. Combining the time-mean states and variances of *D*(*K*_M_)+*D*(*K*_E_), we find that the total dissipation rate of kinetic energies (*D*(*K*_M_)+*D*(*K*_E_)) increased from (2.69±0.20)−(0.63±0.25)/2∼2.38±0.24 W m^−2^ to (2.69±0.20)+(0.63±0.25)/2∼3.01±0.24 W m^−2^ during the past 35 years for NCEP-DOE R2. For ERA-Interim, the total dissipation rate of kinetic energies increased from (2.02±0.16)−(0.18±0.07)/2∼1.93±0.17 W m^−2^ to (2.02±0.16)+(0.18±0.07)/2∼2.11±0.17 W m^−2^ during the past 35 years. Therefore, during the past 35 years the heat engine's efficiency increased from 2.38±0.24/238∼1.0±0.1% to 3.01±0.24/238∼1.3±0.1% for NCEP-DOE R2 and from 1.93±0.17/238∼0.8±0.1% to 2.11±0.17/238∼0.9±0.1% for ERA-Interim. It should be mentioned that the MERRA-2 analyses (Supplementary Fig. 13), which are used to do the validation, also show the linear trends basically consistent with the results from the NCEP-DOE R2 and ERA-Interim data sets.

## Discussion

The Lorenz energy cycle of the global atmosphere and its spatio-temporal variations are investigated with the latest versions of three reanalysis data sets (that is, NCEP-DOE R2 and ERA-Interim, and MERRA-2). Our investigations suggest that the total mechanic energy of the global atmosphere did not significantly change during the past 35 years (1979–2013). However, the eddy energies display significant positive trends especially in the SH. In addition, positive trends are revealed in all conversion terms and generation/dissipation rates of energies during the past 35 years.

This study will help to understand the climate change in a broader perspective. The statistical analyses of the Lorenz energy cycle provide an important constraint on the climate change: the efficiency of Earth's atmosphere as a heat engine increased in response to the climate change (for example, global warming). Recently, there have been discussions of the efficiency of Earth's global atmosphere considered as a thermodynamic heat engine, in which the hydrological cycle was discovered to play an important role^28^,^30^. Our analyses suggest that the dissipation of kinetic energies related to the atmospheric circulation also significantly contributes to the temporal variations of the efficiency of atmospheric heat engine. Climate change triggers intensified eddy and storm activities^44^,^45^,^46^,^47^,^48^,^49^,^59^, which appear as increased eddy energies. The increased eddy energies are accompanied by increased conversion rates, as revealed in this study. The increased conversion rates are further balanced by increased generation/dissipation rates, so that finally the efficiency of the heat engine of the global atmosphere increased during the past 35 years.

The increased eddy energies revealed in this study provide one more piece of evidence for intensifying eddy activities (for example, cyclones and hurricanes) in our climate system^45^,^46^,^47^,^48^,^49^,^50^,^51^,^52^. Our estimates of the generation and dissipation rates of energies also offer important hints to the distribution and variability of the heating/cooling and friction in the global atmosphere, which cannot be measured easily. Finally, the statistical characteristics of the global atmospheric energy cycle are important for the validation of the simulations of climate change, for they constitute further constraints that must be fulfilled.

## Methods

### Theoretical framework and formulation of mechanical energy

The studies of the mechanical energies of Earth's atmosphere were initialized ∼100 years ago. In 1903, Margules^59^ defined the available potential energy as the maximum amount of total potential energy available for conversion into kinetic energy under any adiabatic redistribution of mass. To generalize the definition of available potential energy, Lorenz^1^ introduced another definition for the whole atmosphere, in which the available potential energy was defined as the difference between the total potential energy of the whole atmosphere and the minimum of total potential energy. Using the definition of available potential energy and a common definition of kinetic energy in an atmospheric system, Lorenz further introduced a formulation of atmospheric energies and energy conversions in wind and temperature fields, which is generally knowed as the Lorenz energy cycle^1^. The Lorenz energy cycle was almost immediately used by Philips^2^ in his classical work simulating the general circulation of Earth's atmosphere in a two-level quasi-geostrophic model. Saltzman^18^ extended the formulation in the wave-number domain by employing a Fourier transform so that different scales of motions, including planetary, synoptic and mesoscale circulations, could be examined. In 1964, Oort^19^ reformulated Lorenz's equations of atmospheric energetics in a mixed domain of space and time based on the primitive equations of motion. The formulation by Oort^19^ has been widely used in recent years, because it does not make hydrostatic and geostrophic approximations. The other advantage of Oort's formulation is that it can discriminate between transient eddies (perturbations in time) and stationary eddies (perturbation in space). Peixoto and Oort^5^ further organized and used the formulation to analyse the annual distribution of the mechanical energies with observations. The organized formulation^5^ is used in this study. There are generally two methods of evaluating the Lorenz energy cycle of the global atmosphere—the monthly evaluation and the yearly evaluation^5^,^9^,^33^—in which the eddies are defined as atmospheric processes lasting less than 1 month and 1 year, respectively. In our previous study^9^, we suggested that the large-scale meridional circulation is better resolved in the monthly evaluation than in the yearly evaluation. Therefore, we present the monthly evaluation that characterizes the spatio-temporal characteristics of the Lorenz energy cycle.

### Processing data sets for analysis

The meteorological variables (temperature, winds and geopotential height), which are used in the formulation of the Lorenz energy cycle^5^, come from the latest versions of three reanalysis data sets from the NCEP-DOE R2 (refs 35, 36, 37), the ERA-Interim^38^,^39^,^40^, and the NASA MERRA-2 (refs 41, 42). The corresponding data were downloaded from the websites of the three reanalysis data sets (NCEP-DOE R2, ERA-Interim and MERRA-2) at http://www.esrl.noaa.gov/psd/data/gridded/data.ncep.reanalysis2.html, http://apps.ecmwf.int/datasets/data/interim-full-daily/levtype=sfc/ and http://disc.sci.gsfc.nasa.gov/daac-bin/FTPSubset2.pl. The temporal coverage of the downloaded NCEP-DOE R2 daily data is from 1 January 1979 to 31 December 2013. The NCEP-DOE R2 data have a spatial coverage of global grids at 2.5° × 2.5° (latitude × longitude) resolutions and 17 pressure levels (1,000, 925, 850, 700, 600, 500, 400, 300, 250, 200, 150, 100, 70, 50, 30, 20 and 10 mbar). The downloaded ERA-Interim data have the same temporal coverage as that of the NCEP-DOE R2 data but with different spatial resolutions (1.5° × 1.5° at the latitude-longitude grids and 37 pressure levels from 1 to 1,000 mbar). The downloaded MERRA-2 data have a different temporal coverage (1 January 1980 to 31 December 2013) with varying spatial resolutions. To keep the consistency among the three data sets, we interpolate the ERA-Interim and MERRA-2 data sets to the spatial grids of the NCEP-DOE R2 data set.

### Computing spatial structures and global integrals

The formulation developed by Peixoto and Oort^5^ is applied to the processed data from the three reanalysis data sets (NCEP-DOE R2, ERA-Interim and MERRA-2) to compute the energy components of the Lorenz energy cycle (that is, energies and conversion rates). The mean energies (*P*_M_ and *K*_M_) and the conversion rates associated with the mean energies (*C*(*P*_M_, *P*_M_), *C*(*K*_E_, *K*_M_) and *C*(*P*_M_, *K*_M_)) are computed in a two-dimensional (2D) (latitude × pressure) domain. The eddy energies (*P*_K_ and *K*_K_) and the conversion rate from *P*_E_ to *K*_E_ (*C*(*P*_E_, *K*_E_)) are computed in a three-dimensional (3D) (longitude × latitude × pressure) domain. The 3D energy components are averaged over longitude to get the corresponding quantities in the 2D domain (latitude × pressure). Then we can average the 2D energy components over latitude with a weighting factor of cosine of latitude and integrate over pressure to get the global-average quantities. On the other hand, the 3D energy components can be integrated over pressure first to get these quantities in another 2D (longitude × latitude) domain. Such 2D energy components can be averaged over latitude and longitude to get the global-average quantities too. Finally, the global-average energy components are modified with a multiplication factor^32^, which takes into account the mean mass distribution over the globe (for example, less mass over the mountains). The zonal-mean multiplication factor has a range from 0.94 to 1.00, except for Antarctica, in which the factor varies from 0.75 to 0.90. At the global scale, the generation/dissipation rates in the Lorenz energy cycle should be balanced with the conversion rates and the time derivatives of energies. Therefore, the generation rates of the mean and eddy potential energies (*G*(*P*_M_) and *G*(*P*_E_)), and the dissipation rates of the mean and eddy kinetic energies (*D*(*K*_M_) and *D*(*K*_E_)) are evaluated by balancing the corresponding conversion rates and the time derivatives of energies based on the flow chart shown in Supplementary Fig. 1. The conversion rates (Fig. 2) are much larger than the time derivatives of the corresponding energies (Supplementary Fig. 9). However, we keep the time derivatives of energies in the computation of generation/dissipation rates in order to make the results as precise as possible. Here we take *D*(*K*_E_) as an example to explain the computations of generation/dissipation rates. Based on the flow chart shown in Supplementary Fig. 1, the time derivative of the eddy kinetic energy can be expressed as *dK*_E_/*dt*=*C*(*P*_E_, *K*_E_)−*C*(*P*_E_, *K*_M_)−*D*(*K*_E_). Thus, we have *D*(*K*_E_)=*C*(*P*_E_, *K*_E_)−*C*(*P*_E_, *K*_M_)−*dK*_E_/*dt*. The two conversion rates are computed based on the reanalysis data sets (Fig. 2). The time derivative *dK*_E_/*dt* can be computed from the time series of the eddy kinetic energy (Fig. 1), which is shown in Supplementary Fig. 9. Therefore, *D*(*K*_E_) can be computed from *C*(*P*_E_, *K*_E_), *C*(*K*_E_, *K*_M_) and *dK*_E_/*dt*. Likewise, we can compute *G*(*P*_M_), *G*(*P*_E_) and *D*(*K*_M_) by the corresponding conversion rates and the time derivatives of energies.

### Analysing the linear trends of the energy components

The analyses of the linear trends of the Lorenz energy cycle are based on the time series of the computed energy components. Before calculating the linear trends of the energy components, we process the time series of the energy components as follows: First, the time-mean values and seasonal cycles are removed. Second, a low-pass filter is applied to remove the high-frequency variations with time periods <1 year^60^. Finally, El Nino-Southern Oscillation signals are removed by a regression method based on the Nino 3.4 index to emphasize the long-term trends. Then we calculate the linear trends of the processed time series of the energy components and the corresponding uncertainties by the least-squares method^43^. The confidence levels (that is, probabilities) of the linear trends are estimated by Student's *t*-statistics^61^. The *t*-statistic is defined by 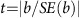, where *b* is the linear trend and *SE*(*b*) is the s.e. of the linear trend *b*. We can compute *SE*(*b*) by 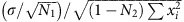, where *σ* is the s.d. of the data, *N*_1_ is the number of degrees of freedom of the data, *N*_2_ is the length of the data set and *x*_*i*_ is the time series of data after subtracting the time mean. The number of degrees of freedom *N*_1_ is estimated by an equation^62^ suggested by 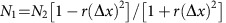, where *r*(Δ*x*) is the autocorrelation corresponding to a lag of time interval Δ*x*. The confidence level of a linear trend is estimated by comparing *t* with a certain value *t*_0_, which can be found from the *t*-distribution table^43^. The analyses of the linear trends of the energy components in the Lorenz energy cycle are conducted not only for the global-average quantities but also for the quantities in the 2D (longitude × latitude or latitude × pressure) domain.

### Data availability

The original data from the two reanalysis centres (NCEP-DOE R2 and ERA-Interim) are publicly available and can be freely downloaded from the corresponding websites of the three reanalysis data sets (NCEP-DOE R2, ERA-Interim and MERRA-2) at http://www.esrl.noaa.gov/psd/data/gridded/data.ncep.reanalysis2.html, http://apps.ecmwf.int/datasets/data/interim-full-daily/levtype=sfc/ and http://disc.sci.gsfc.nasa.gov/daac-bin/FTPSubset2.pl. The authors declare all data supporting the results in the article are available. The data for the most important scientific results (for example, spatio-temporal characteristics of the Lorenz energy cycle) are presented in the article and the Supplementary Information. The processed data sets and the complete analyses of the spatial-temporal characteristics of the Lorenz energy cycle are available on request from L.L. In addition, the codes for computing and analysing the Lorenz energy cycle are available on request from L.L.

## Additional information

**How to cite this article:** Pan, Y. *et al*. Earth's changing global atmospheric energy cycle in response to climate change. *Nat. Commun.* **8,** 14367 doi: 10.1038/ncomms14367 (2017).

**Publisher's note**: Springer Nature remains neutral with regard to jurisdictional claims in published maps and institutional affiliations.

## Supplementary Material

Supplementary Information Supplementary Tables and Supplementary Figures

## Figures and Tables

**Table 1:** Linear trends and the corresponding confidence levels of energy components of the Lorenz energy cycle of the global atmosphere 1979–2013.

Energy component	NCEP-DOE R2		ERA-Interim	
	**Trend**	**Confidence**	**Trend**	**Confidence**
*P*_M_ (J m^−2^ per year)	−655.5±1662.6	<70%	−955.6±1579.3	<70%
*P*_E_ (J m^−2^ per year)	755.5±391.5	96.4%	571.6±433.2	90.3%
*K*_M_ (J m^−2^ year^−1^)	83.5±423.2	<70%	378.8±485.6	<70%
*K*_E_ (J m^−2^ per year)	1259.5±480.0	99.2%	362.1±304.5	87.8%
Total *E* (J m^−2^ per year)	1143.1±2066.9	<70%	396.9±1843.6	<70%
*C*(*P*_M_, *P*_E_) (10^−3^ W m^−2^ per year)	2.2±1.2	96.0%	2.0±1.2	94.1%
*C*(*P*_E_, *K*_E_) (10^−3^ W m^−2^ per year)	11.2±4.9	95.8%	2.7±1.5	96.0%
*C*(*K*_E_, *K*_M_) (10^−3^ W m^−2^ per year)	0.8±0.5	91.2%	0.6±0.5	86.5%
*C*(*P*_M_, *K*_M_) (10^−3^ W m^−2^ per year)	7.6±3.6	95.3%	2.2±1.0	97.4%
*G*(*P*_M_) (10^−3^ W m^−2^ per year)	9.2±4.8	94.8%	3.8±2.0	93.4%
*G*(*P*_E_) (10^−3^ W m^−2^ per year)	8.9±4.8	93.4%	1.0±0.8	81.3%
*D*(*K*_M_) (10^−3^ W m^−2^ per year)	7.6±4.4	92.3%	3.0±1.4	96.5%
*D*(*K*_E_) (10^−3^ W m^−2^ per year)	10.3±5.6	94.4%	2.0±1.5	87.2%

ERA-Interim, European Centre for Medium-Range Weather Forecasts Re-Analysis Interim; NCEP-DOE R2, National Centers of Environmental Prediction and the Department of Energy Reanalysis II.

Note: The corresponding confidence levels (that is, the probabilities of linear trends with a non-zero slope) are estimated by the Student's *t*-statistics (please see Methods). The linear trends with confidence levels >90%, between 80 and 90%, and <70% are highlighted in blue, yellow and black colours, respectively.

## References

[b1] Lorenz E. N. Available potential energy and the maintenance of the general circulation. Tellus 7, 157–167 (1955).

[b2] Phillips N. A. The general circulation of the atmosphere: a numerical experiment. Quart. J. R. Met. Soc. 82, 123–164 (1956).

[b3] Krueger A. F., Winston J. S. & Haines D. A. Computation of atmospheric energy and its transformation for the Northern Hemisphere for a recent five-year period. Mon. Weather Rev. 93, 227–238 (1965).

[b4] Wiin-Nielsen A. On the annual variation and spectral distribution of atmospheric energy. Tellus 19, 540–559 (1967).

[b5] Peixóto J. P. & Oort A. H. The annual distribution of atmospheric energy on a planetary scale. J. Geophys. Res. 79, 2149–2159 (1974).

[b6] Oort A. H. & Peixóto J. P. The annual cycle of the energetics of the atmosphere on a planetary scale. J. Geophys. Res. 79, 2705–2719 (1974).

[b7] Oort A. H. & Peixóto J. P. On the variability of the atmospheric energy cycle within a 5-year period. J. Geophys. Res. 81, 3643–3659 (1976).

[b8] Hu Q., Tawaye Y. & Feng S. Variations of the Northern Hemisphere atmospheric energetics: 1948–2000. J. Clim. 17, 1975–1986 (2004).

[b9] Li L., Ingersoll A. P., Jiang X., Feldman D. & Yung Y. L. Lorenz energy cycle of the global atmosphere based on reanalysis data sets. Geophys. Res. Lett. 34, L16813 (2007).

[b10] Li L., Jiang X., Chahine M. T., Wang J. & Yung Y. L. The mechanical energies of the global atmosphere in El Niño and La Niña Years. J. Atmos. Sci. 68, 3072–3078 (2011).

[b11] Steinheimer M., Hantel M. & Bechtold P. Convection in Lorenz's global energy cycle with the ECMWF model. Tellus 60, 1001–1022 (2008).

[b12] Kim Y. H. & Kim M. K. Examination of the global lorenz energy cycle using MERRA and NCEP-reanalysis 2. Clim. Dyn. 40, 1499–1513 (2013).

[b13] Huang J. & McElroy M. B. Contributions of the Hadley and Ferrel circulations to the energetics of the atmosphere over the past 32 years. J. Clim. 27, 2656–2666 (2014).

[b14] Huang J. & McElroy M. B. Thermodynamic disequilibrium of the atmosphere in the context of global warming. Clim. Dyn. 45, 3513–3525 (2015).

[b15] Schubert G. & Mitchell J. L. in *Comparative Climatology of Terrestrial Planets* (eds Mackwel, S. J. .) 181–191 (Univ. Arizona Press, 2013).

[b16] Lucarini V., Pascale S., Boschi R., Kirk E. & Iro N. Habitability and multistability in Earth-like planets. Astron. Nachr. 334, 576–588 (2013).

[b17] Tabataba-Vakili F. . A Lorenz/Boer energy budget for the atmosphere of Mars from a ‘reanalysis' of spacecraft observations. Geophys. Res. Lett. 42, 8320–8327 (2015).

[b18] Saltzman B. Equations governing the energetics of the larger scales of atmospheric turbulence in the domain of wave number. J. Meteor. 14, 513–523 (1957).

[b19] Oort A. H. On estimates of the atmospheric energy cycle. Mon. Weather Rev. 92, 483–499 (1964).

[b20] Darkow G. L. Total energy environment of severe storms. Bull. Am. Meteorol. Soc. 48, 495–495 (1967).

[b21] Lorenz E. N. Available energy and the maintenance of a moist circulation. Tellus 30, 15–31 (1978).

[b22] Moncrieff M. W. & Miller M. J. The dynamics and simulation of tropical cumulonimbus and squall lines. Quarter. J. R. Meteorol. Soc. 102, 373–394 (1976).

[b23] Randall D. A. & Wang J. The moist available energy of a conditionally unstable atmosphere. J. Atmos. Sci. 49, 240–255 (1992).

[b24] Blanchard D. O. Assessing the vertical distribution of convective available potential energy. Weather Forecast. 13, 870–877 (1998).

[b25] Lucarini V. Thermodynamic efficiency and entropy production in the climate system. Phys. Rev. E 80, 021118 (2009). 10.1103/PhysRevE.80.021118 19792088

[b26] Lucarini V. & Ragone F. Energetics of climate models: net energy balance and meridional enthalpy transport. Rev. Geophys. 49, 1–29 (2011).

[b27] Storch J. S. V. . An estimate of the Lorenz energy cycle for the world ocean based on the STORM/NCEP simulation. J. Phys. Oceanogr. 42, 2185–2205 (2012).

[b28] Pauluis O. & Dias J. Satellite estimates of precipitation-induced dissipation in the atmosphere. Science 335, 953–956 (2012). 22363004 10.1126/science.1215869

[b29] Lucarini V. . Mathematical and physical ideas for climate science. Rev. Geophys. 52, 809–859 (2014).

[b30] Laliberté F. . Constrained work output of the moist atmospheric heat engine in a warming climate. Science 347, 540–543 (2015). 25635098 10.1126/science.1257103

[b31] Huang J. A climate-friendly energy future: prospects for wind. Doctoral Dissert. (2014).

[b32] Huang J. & McElroy M. B. A 32-year perspective on the origin of wind energy in a warming climate. Renew. Energy 77, 482–492 (2015).

[b33] Oort A. in *Global Atmospheric Circulation Statistics, 1958–1973*. 180–226 (National Oceanic and Atmospheric Administration, U.S. GPO, Washington, DC, 1983).

[b34] Peixoto J. P. & Oort A. H. Physics of Climate American Institute of Physics (1992).

[b35] Kalnay E. & Kanamitsu M. The NCEP/NCAR 40-year reanalysis project. Bull. Am. Meteorol. Soc. 77, 437–471 (1996).

[b36] Kistler R., Collins W. & Saha S. The NCEP-NCAR 50-year reanalysis: monthly means CD-ROM and documentation. Bull. Am. Meteorol. Soc. 82, 247–267 (2001).

[b37] Kanamitsu M. & Ebisuzaki W. NCEP–DOE AMIP-II Reanalysis (R-2). Bull. Am. Meteorol. Soc. 83, 1631–1643 (2002).

[b38] Uppala S. M. . The ERA-40 re-analysis. Quart. J. R. Meteor. Soc. 131, 2961–3012 (2005).

[b39] Berrisford P. . *The ERA-Interim Archive Version 2.0, ERA Report Series 1*. 13177 (ECMWF, Shinfield Park, Reading, UK, 2011).

[b40] Dee D. P. . The ERA-Interim reanalysis: configuration and performance of the data assimilation system. Quart. J. R. Meteorol. Soc. 137, 553–597 (2011).

[b41] Rienecker M. M. . MERRA: NASA's modern-era retrospective analysis for research and applications. J. Clim. 24, 3624–3648 (2011). 10.1175/JCLI-D-16-0758.1 PMC6999672 32020988

[b42] Molod A., Takacs L., Suarez M. & Bacmeister J. Development of the GEOS-5 atmospheric general circulation model: evolution from MERRA to MERRA2. Geosci. Model Dev. 8, 1339–1356 (2015).

[b43] Belington P. R. & Robinson D. K. Data Reduction and Error Analysis for the Physical Sciences 3rd Edn McGraw-Hill (2003).

[b44] Davi N. & Jacoby G. Extension of drought records for Central Asia using tree rings: West-Central Mongolia. J. Clim. 19, 288–299 (2006).

[b45] Emanuel K. A. The dependence of hurricane intensity on climate. Nature 326, 483–485 (1987).

[b46] Emanuel K. Increasing destructiveness of tropical cyclones over the past 30 years. Nature 436, 686–688 (2005). 16056221 10.1038/nature03906

[b47] Knutson T. R. & Tuleya R. E. Impact of CO2-induced warming on simulated hurricane intensity and precipitation: sensitivity to the choice of climate model and convective parameterization. J. Clim. 17, 3477–3495 (2004).

[b48] Webster P. J., Holland G. J., Curry J. A. & Chang H. R. Changes in tropical cyclone number, duration, and intensity in a warming environment. Science 309, 1844–1846 (2005). 16166514 10.1126/science.1116448

[b49] Emanuel K., Sundararajan R. & Williams J. Hurricanes and global warming: results from downscaling IPCC AR4 simulations. Bull. Am. Meteorol. Soc. 89, 347–367 (2008).

[b50] Knutson T. R. . Tropical cyclones and climate change. Nat. Geosci. 3, 157–163 (2010).

[b51] Fischer-Bruns I., von S. H., Gonzales-Rouco J. F. & Zorita E. Modelling the variability of midlatitude storm activity on decadal to century time scales. Clim. Dyn. 25, 461–476 (2005).

[b52] Simmonds I. Modes of atmospheric variability over the Southern Ocean. J. Geophys. Res. Ocean 108, (2003).

[b53] Mitas C. M. & Clement A. Has the Hadley cell been strengthening in recent decades? Geophys. Res. Lett. 32, (2005).

[b54] Seidel D. J., Fu Q., Randel W. J. & Reichler T. J. Widening of the tropical belt in a changing climate. Nat. Geosci. 1, 21–24 (2008).

[b55] Liu J., Song M., Hu Y. & Ren X. Changes in the strength and width of the Hadley Circulation since 1871. Clim. Past 8, 1169–1175 (2012).

[b56] Gulev S. K., Zolina O. & Grigoriev S. Extratropical cyclone variability in the Northern Hemisphere winter from the NCEP/NCAR reanalysis data. Clim. Dyn. 17, 795–809 (2001).

[b57] McCabe G. J., Clark M. P. & Serreze M. C. Trends in Northern Hemisphere surface cyclone frequency and intensity. J. Clim. 14, 2763–2768 (2001).

[b58] Leibensperger E. M., Mickley L. J. & Jacob D. J. Sensitivity of US air quality to mid-latitude cyclone frequency and implications of 1980–2006 climate change. Atmos. Chem. Phys. 8, 7075–7086 (2008).

[b59] Margules M. Uber die Energie der Sturme. Jahrb. Zentralanst. Meteorol. 40, 1–26 (1903) (Translated from German by C. Abbe, *Smithson. Misc. Collect*. **51**, 553–595, 1910).

[b60] Jiang X. . Quasi-biennial oscillation and quasi-biennial oscillation-annual beat in the tropical total column ozone: a two-dimensional model simulation. J. Geophys. Res. 109, (2004).

[b61] Box G. E. P., Hunter J. S. & Hunter W. G. Statistical for Experiments 2nd edn John Wiley & Sons Inc. (2005).

[b62] Bretherton C. S., Widmann M., Dymnikov V. P., Wallace J. M. & Blade I. The effective number of spatial degrees of freedom of a time-varying field. J. Clim. 12, 1990–2009 (1999).

