# Supplementary material for: Earth's changing global atmospheric energy cycle in response to climate change
Source: Nat Commun. 2017 Jan 24;8:14367. doi: 10.1038/ncomms14367 (PMC5286208; doi:10.1038/ncomms14367)
Supplement: Supplementary Information — Supplementary Tables and Supplementary Figures [file ncomms14367-s1.pdf]

Supplementary Table 1: The time-mean, temporal variance, and varied percentage of the energy components of the Lorenz energy cycle of the global atmosphere over the past 35 years (1979-2013).

Energy Component	NCEP-DOE R2			ERA-Interim		
	Mean	Variance	Percentage	Mean	Variance	Percentage
P_M (10^5 J m^{-2})	42.30±2.49	-0.23±0.58	0.5±1.4%	45.44±2.70	-0.33±0.56	-0.7±1.2%
P_E (10^5 J m^{-2})	5.03±0.59	0.26±0.14	5.2±2.7%	6.56±0.89	0.20±0.15	3.0±2.3%
K_M (10^5 J m^{-2})	7.68±0.56	0.03±0.15	0.4±2.0%	7.82±0.58	0.13±0.17	1.7±2.2%
K_E (10^5 J m^{-2})	6.31±0.53	0.44±0.17	7.0±2.8%	6.64±0.51	0.13±0.11	2.0±1.7%
Total E (10^5 J m^{-2})	61.32±3.44	0.40±0.72	0.7±1.2%	66.46±3.88	0.14±0.65	0.2±1.0%
$C(P_M, P_E)$ (W m^{-2})	1.75±0.17	0.08±0.04	4.6±2.3%	1.73±0.20	0.07±0.04	4.0±2.4%
$C(P_E, K_E)$ (W m^{-2})	2.47±0.16	0.39±0.17	15.8±7.0%	1.91±0.16	0.10±0.05	5.2±2.7%
$C(K_E, K_M)$ (W m^{-2})	0.35±0.06	0.03±0.02	8.6±5.9%	0.20±0.06	0.02±0.02	10.0±10.4%
$C(P_M, K_M)$ (W m^{-2})	0.22±0.11	0.27±0.13	122.7±85.2%	0.11±0.09	0.08±0.04	72.7±59.7%
$G(P_M)$ (W m^{-2})	1.97±0.18	0.32±0.17	16.2±8.8%	1.84±0.16	0.13±0.07	7.1±3.9%
$G(P_E)$ (W m^{-2})	0.72±0.19	0.31±0.17	43.1±26.2%	0.18±0.16	0.04±0.03	22.2±25.8%
$D(K_M)$ (W m^{-2})	0.57±0.12	0.27±0.15	47.4±28.1%	0.31±0.09	0.11±0.05	35.5±19.1%
$D(K_E)$ (W m^{-2})	2.12±0.16	0.36±0.20	17.0±9.1%	1.71±0.13	0.07±0.05	4.1±2.9%

Note: The time-mean values are computed by averaging the energy components over the past 35 years. The error-bars of the time-mean values stand for the standard deviations of the time series of the energy components. The temporal variances are computed by multiplying the linear trends of energy components (Table 1) by the 35-year time period. The percentages are the ratios between the temporal variances and the time-mean values. The error-bars of the percentages are estimated by using the standard formulae for propagation of uncertainty: if we have a±da and b±db, then c = a/b has error-bar dc, where $(dc/c)^2 = (da/a)^2 + (db/b)^2$. The different colors come from the colors in Table 1 in the main text.

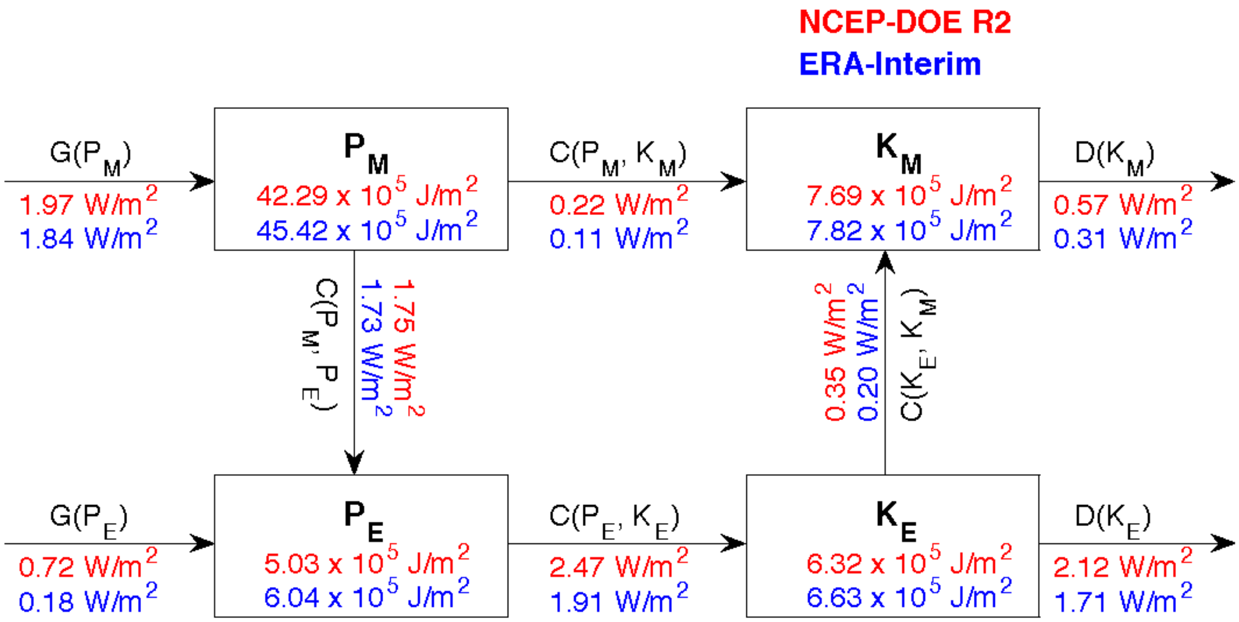

Supplementary Figure 1: Time-mean picture of the Lorenz energy cycle for the global atmosphere. The mechanical energies (i.e., the mean available potential energy P_M, the eddy available potential energy P_E, the mean kinetic energy K_M, and the eddy kinetic energy K_E) and the conversion rates among different energies (i.e., $C(P_M, P_E)$, $C(P_E, K_E)$, $C(K_E, K_M)$, and $C(P_M, K_M)$) are calculated from the monthly evaluation of the NCEP-DOE R2 (red numbers) and ERA-Interim (blue numbers) data sets during the past 35 years (1979-2013). The generation rates of the mean and eddy available potential energies ($G(P_M)$ and $G(P_E)$) and the dissipation rates of the mean and eddy kinetic energies ($D(K_M)$ and $D(K_E)$) are evaluated from the corresponding conversion rates and time derivatives of energies. The 35-year cycle can be thought one of the most reliable pictures for the climatological Lorenz energy cycle.

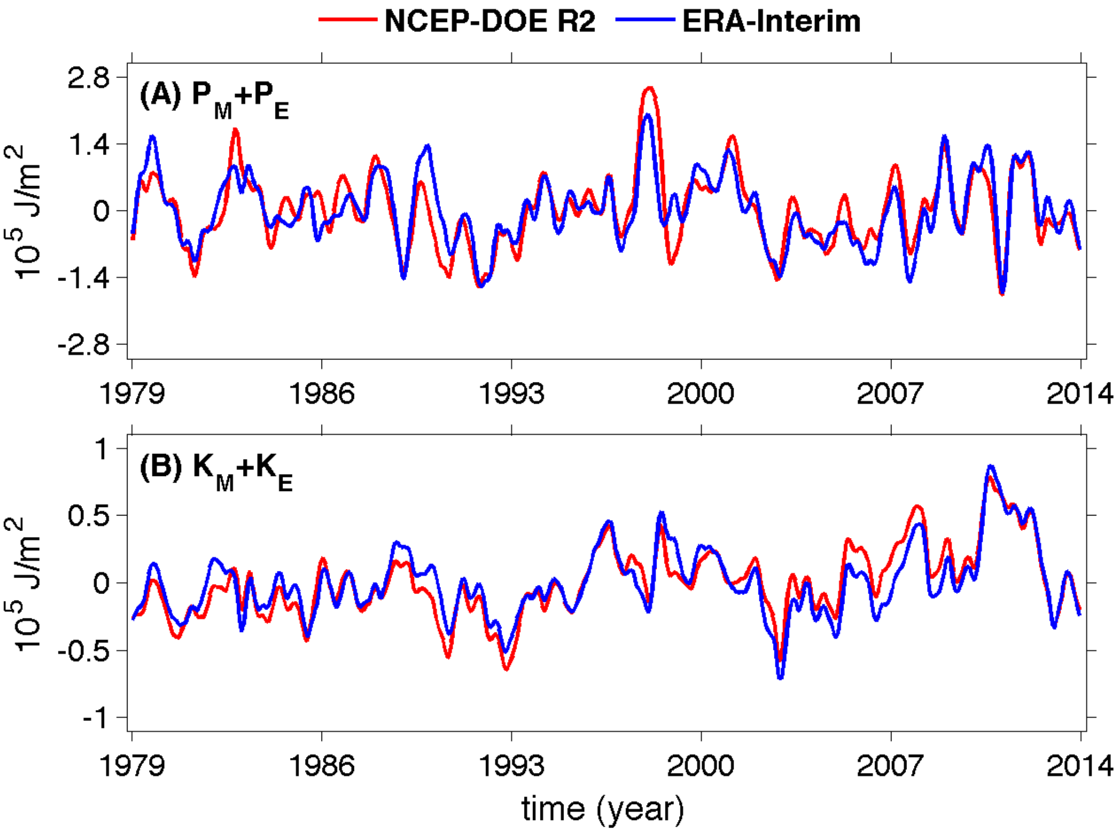

Supplementary Figure 2: Time series of the total available potential energy and kinetic energy. The analyses are based on the monthly evaluation of the NCEP-DOE R2 (red slines) and ERA-Interim (blue lines) data sets during the period of 1979-2013. (A) The total available potential energy ($P_M + P_E$). The linear trends (confidence levels) of the total available potential energy are 100.0±1713.3 J m^{-2} year^{-1} (<70%) and -384.0±1502.9 J m^{-2} year^{-1} (<70%) for the NCEP-DOE R2 and ERA-Interim, respectively. (B) The total kinetic energy ($K_M + K_E$). The linear trends (confidence levels) of the total kinetic energy are 943.3±842.8 J m^{-2} year^{-1} (90.8%) and 780.9±693.3 J m^{-2} year^{-1} (86.5%) for the NCEP-DOE R2 and ERA-Interim, respectively.

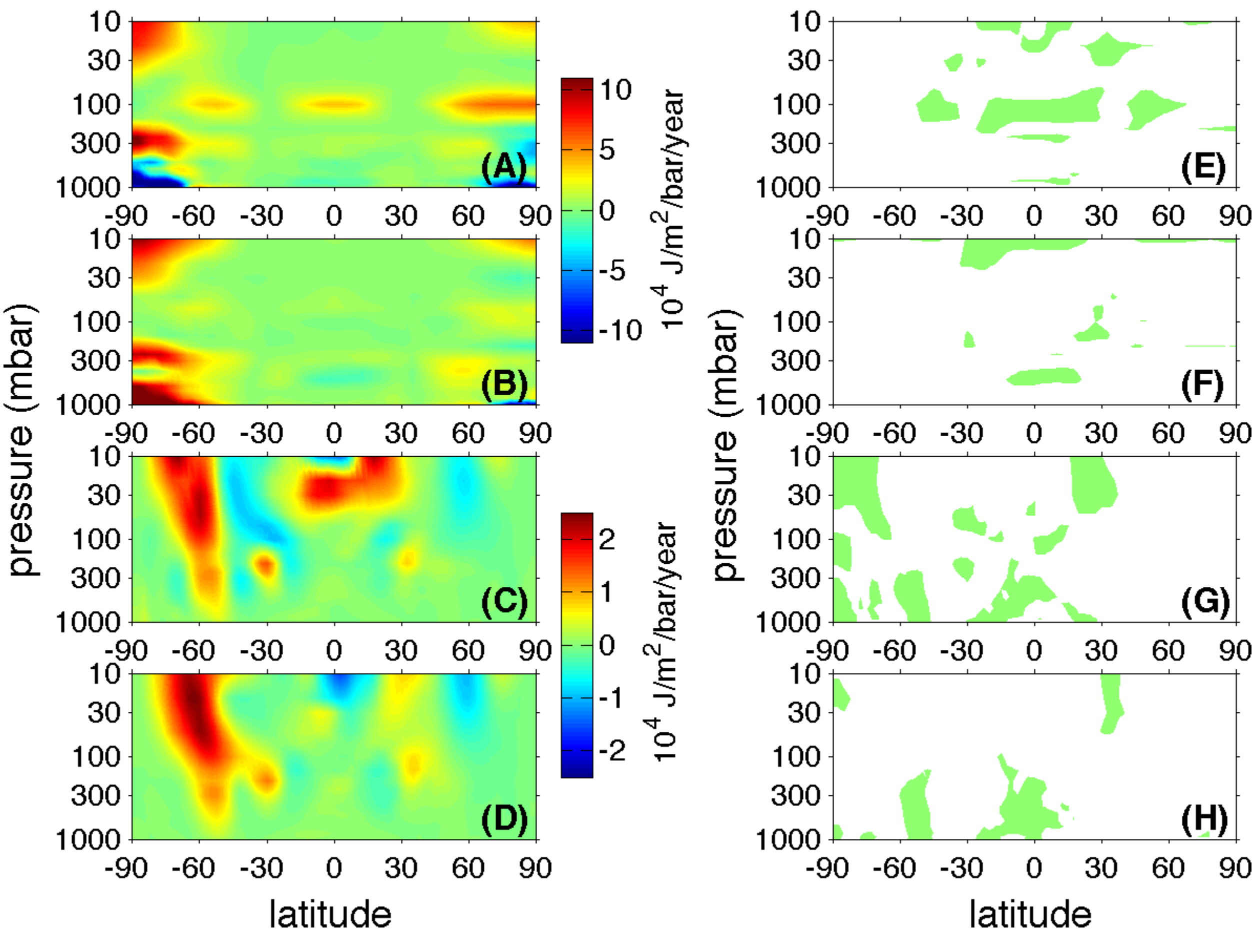

Supplementary Figure 3: Distribution of the linear trends of P_M and K_M in the latitude-pressure cross-section. All panels are plotted with MATLAB Version R2014a. Quantities are averaged in the longitudinal direction to obtain two-dimensional latitude-pressure cross sections. A linear trend and the corresponding confidence level are calculated for each grid-point in the 2-D latitude-pressure cross section. (A) Linear trend of the mean available potential energy (P_M) from the NCEP-DOE R2. (B) Linear trend of P_M from the ERA-Interim. (C) Linear trend of the mean kinetic energy (K_M) from the NCEP-DOE R2. (D) Linear trend of K_M from the ERA-Interim. The confidence levels for the linear trends in panels A, B, C, and D are shown in panels E, F, G, and H, respectively. Areas in which the linear trends (positive or negative) have confidence levels larger than 90% are shown in green color in panels E, F, G, and H.

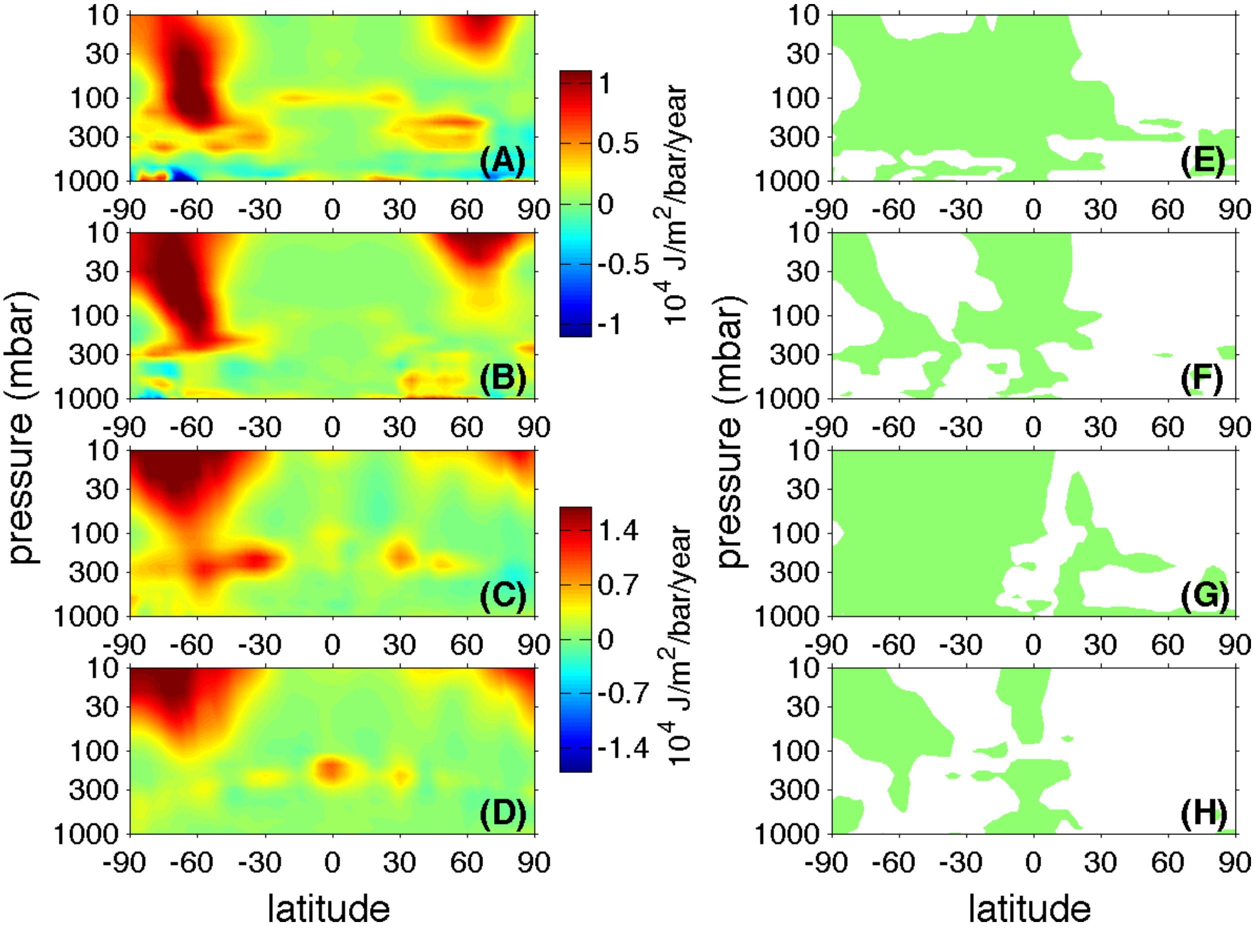

Supplementary Figure 4: Distribution of the linear trends of P_E and K_E in the latitude-pressure cross-section. All panels are plotted with MATLAB Version R2014a. Quantities are averaged in the longitudinal direction to obtain two-dimensional latitude-pressure cross sections. A linear trend and the corresponding confidence level are calculated for each grid-point in the 2-D latitude-pressure cross section. (A) Linear trend of the eddy available potential energy (P_E) from the NCEP-DOE R2. (B) Linear trend of P_E from the ERA-Interim. (C) Linear trend of the eddy kinetic energy (K_E) from the NCEP-DOE R2. (D) Linear trend of K_E from the ERA-Interim. The confidence levels for the linear trends in panels A, B, C, and D are shown in panels E, F, G, and H, respectively. Areas in which the linear trends (positive or negative) have confidence levels larger than 90% are shown in green color in panels E, F, G, and H.

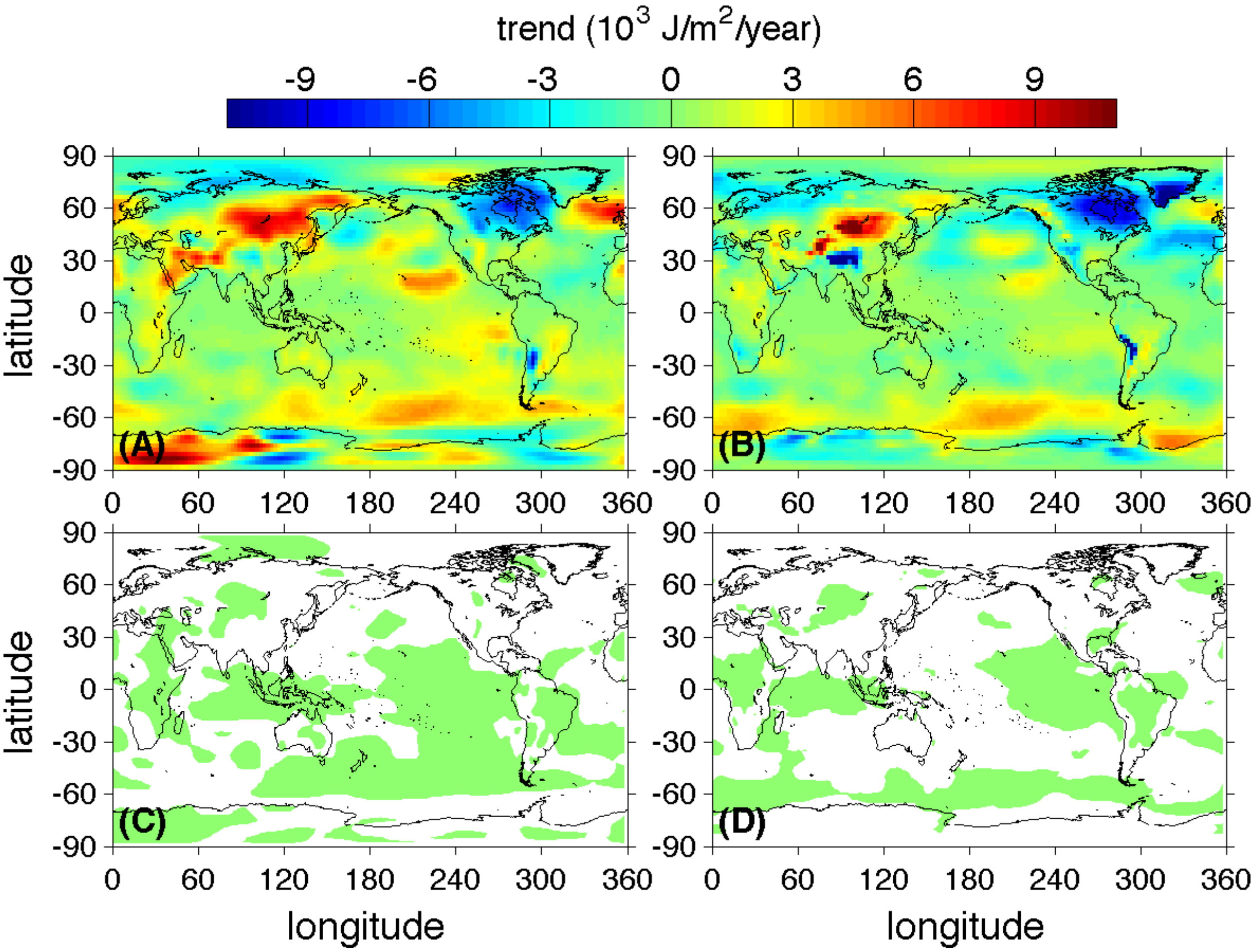

Supplementary Figure 5: Global distribution of the linear trends of the eddy available potential energy (P_E). All panels are plotted with MATLAB Version R2014a. The coastline of the world map was obtained by the National Center for Atmospheric Research (NCAR) with the NCAR Command Language (http://www.ncl.ucar.edu/Applications/mapoutlines.shtml). The 3-D eddy available potential energy is integrated in the vertical direction to get maps in latitude and longitude. A linear trend and the corresponding confidence level are calculated for each grid-point on the maps. (A) Linear trend of the eddy available potential energy (P_E) from the NCEP-DOE R2. (B) Linear trend of P_E from the ERA-Interim. (C) Confidence level for the linear trend of P_E from the NCEP-DOE R2. (D) Confidence level for the linear trend of P_E from the ERA-Interim. Areas in which the linear trends (positive or negative) have confidence levels larger than 90% are shown in green color.

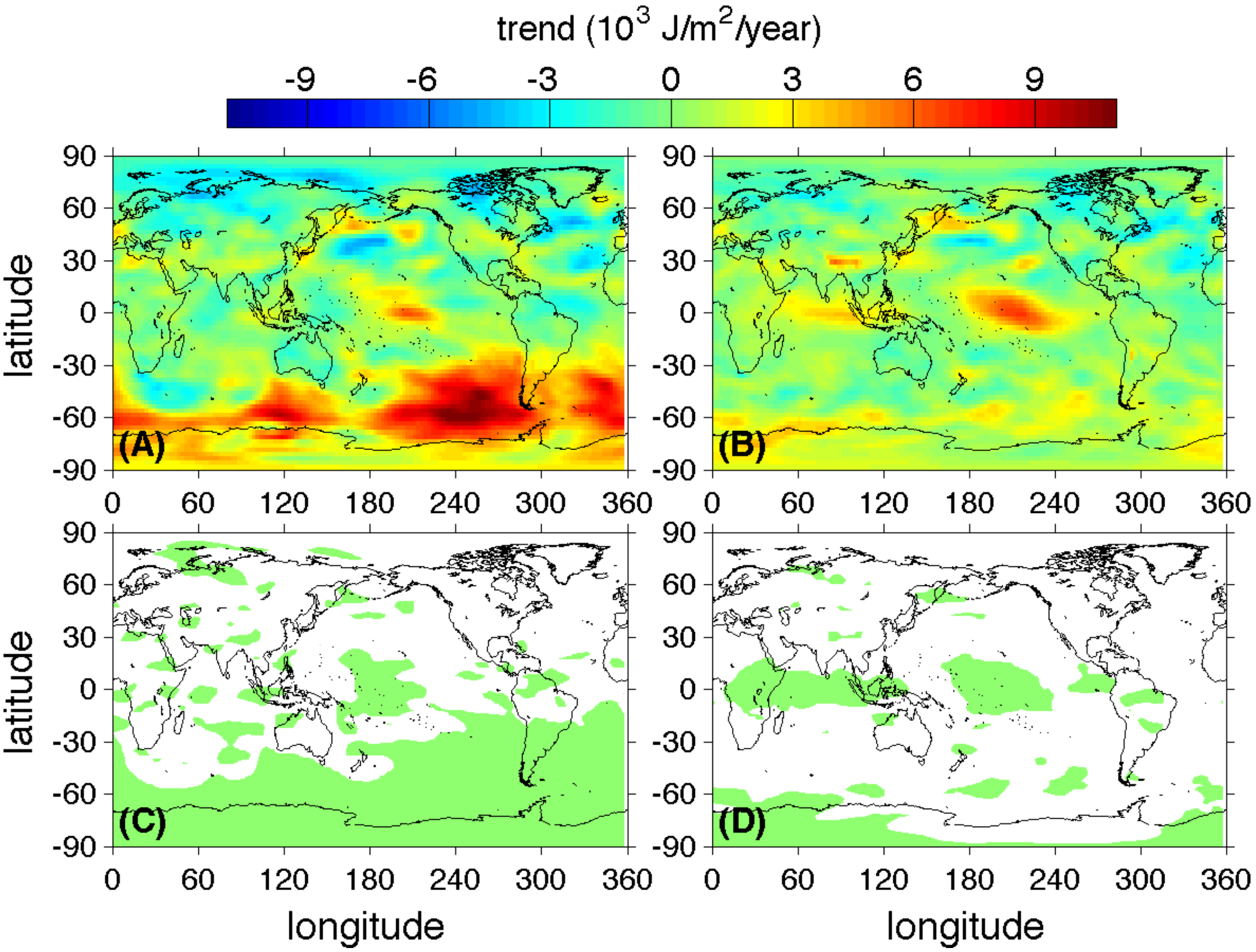

Supplementary Figure 6: Global distribution of the linear trends of the eddy kinetic energy (K_E**).** All panels are plotted with MATLAB Version R2014a. The coastline of the world map was obtained by the National Center for Atmospheric Research (NCAR) with the NCAR Command Language (http://www.ncl.ucar.edu/Applications/mapoutlines.shtml). The 3-D eddy available potential energy is integrated in the vertical direction to get maps in latitude and longitude. A linear trend and the corresponding confidence level are calculated for each grid-point on the maps. (A) Linear trend of the eddy kinetic energy (K_E) from the NCEP-DOE R2. (B) Linear trend of K_E from the ERA-Interim. (C) Confidence level for the linear trend of K_E from the NCEP-DOE R2. (D) Confidence level for the linear trend of K_E from the ERA-Interim. Areas in which the linear trends (positive or negative) have confidence levels larger than 90% are shown in green color.

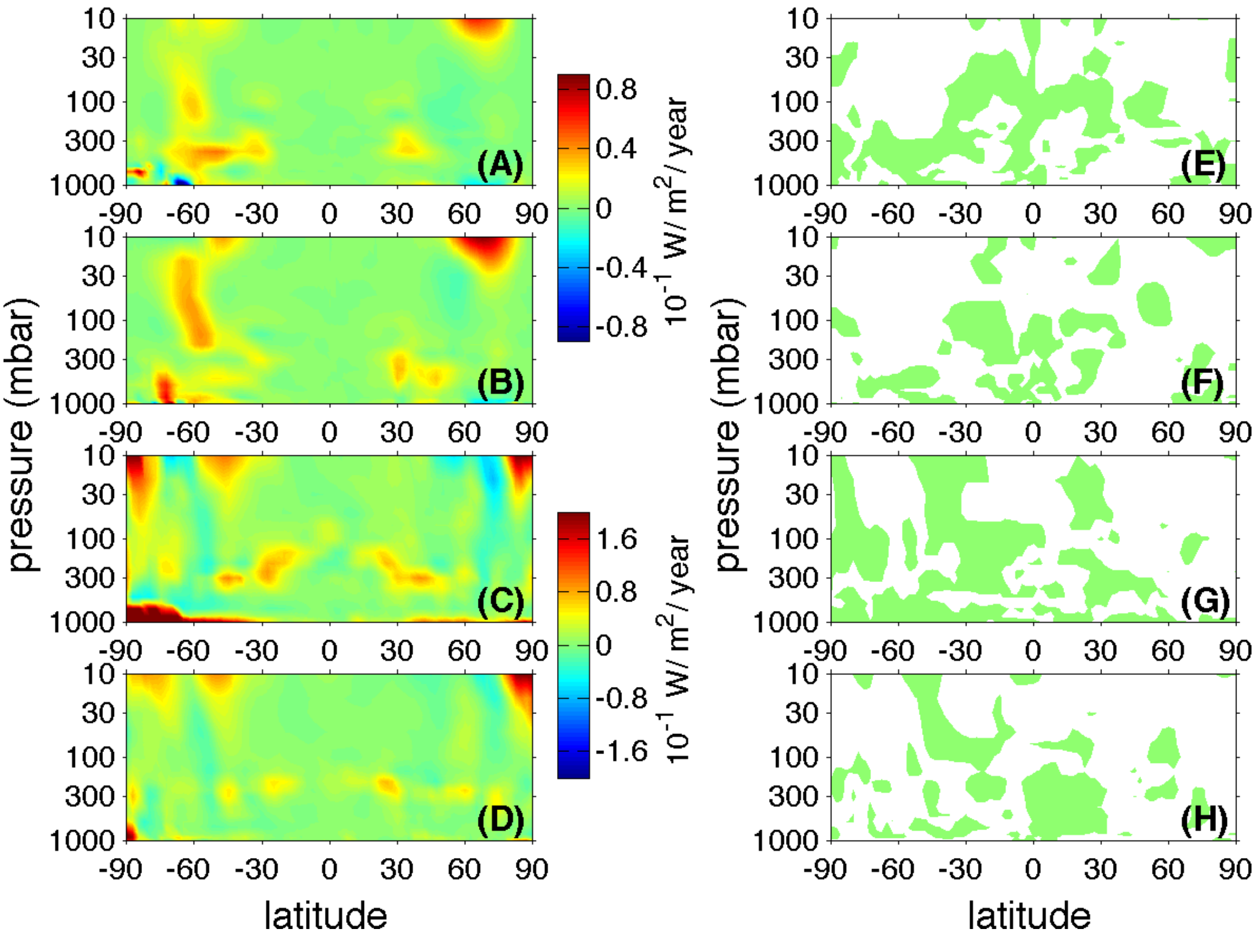

Supplementary Figure 7: Distribution of the linear trends of $C(P_M, P_E)$ and $C(P_E, K_E)$ in the latitude-pressure cross-section. All panels are plotted with MATLAB Version R2014a. A linear trend and the corresponding confidence level are calculated for each grid-point in the 2-D latitude-pressure cross section. (A) Linear trend of the conversion rate between the mean available potential energy and the eddy available potential energy $C(P_M, P_E)$ from the NCEP-DOE R2. (B) Linear trend of $C(P_M, P_E)$ from the ERA-Interim. (C) Linear trend of the conversion rate between the eddy available potential energy and the eddy kinetic energy $C(P_E, K_E)$ from the NCEP-DOE R2. (D) Linear trend of $C(P_E, K_E)$ from the ERA-Interim. The confidence levels for the linear trends in panels A, B, C, and D are shown in panels E, F, G, and H, respectively. Areas in which the linear trends (positive or negative) have confidence levels larger than 90% are shown in green color in panels E, F, G, and H.

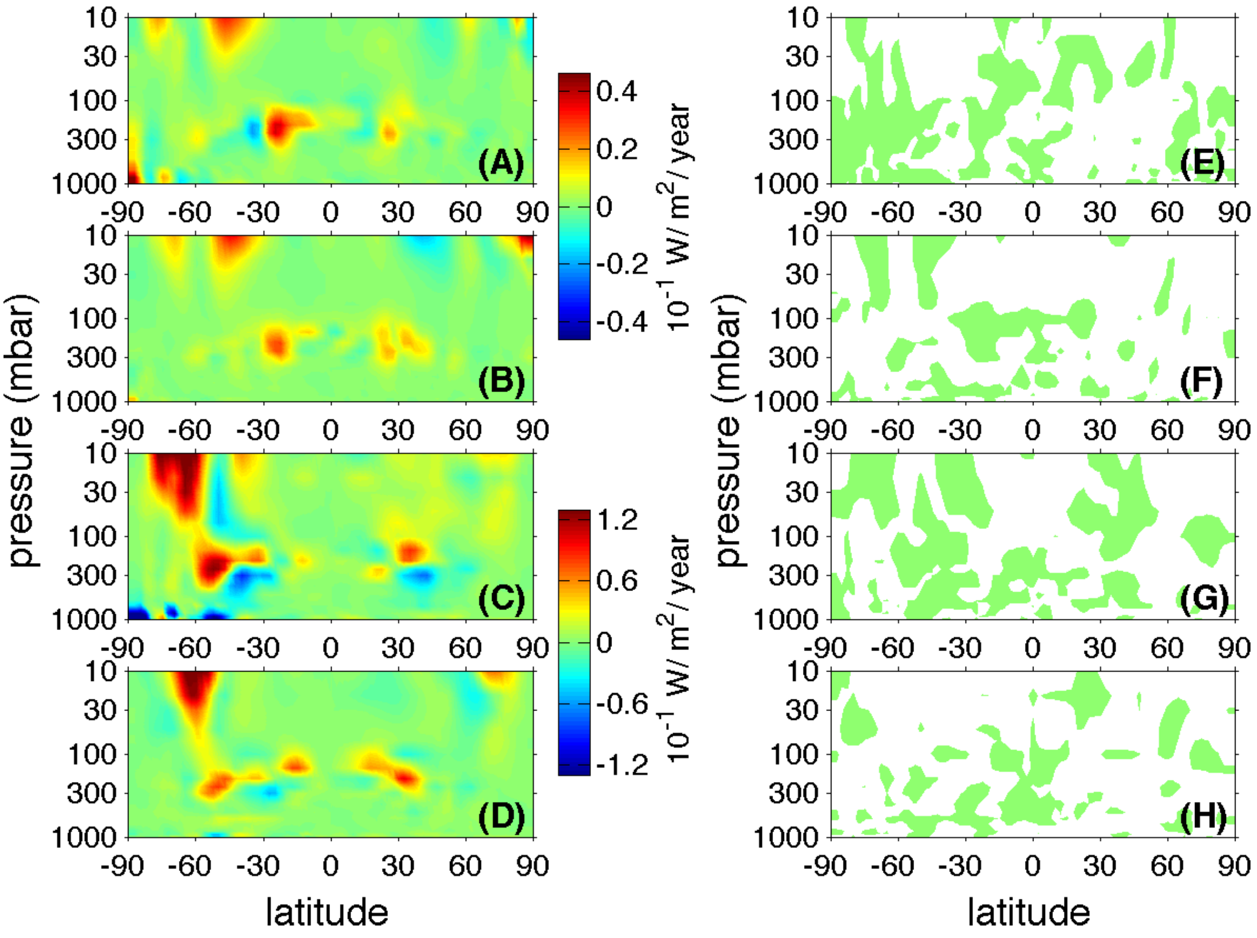

Supplementary Figure 8: Distribution of the linear trends of $C(K_E, K_M)$ and $C(P_M, K_M)$ in the latitude-pressure cross-section. All panels are plotted with MATLAB Version R2014a. A linear trend and the corresponding confidence level are calculated for each grid-point in the 2-D latitude-pressure cross section. (A) Linear trend of the conversion rate between the eddy kinetic energy and the mean kinetic energy $C(K_E, K_M)$ from the NCEP-DOE R2. (B) Linear trend of $C(K_E, K_M)$ from the ERA-Interim. (C) Linear trend of the conversion rate between the mean available potential energy and the mean kinetic energy $C(P_M, K_M)$ from the NCEP-DOE R2. (D) Linear trend of $C(P_M, K_M)$ from the ERA-Interim. The confidence levels for the linear trends in panels A, B, C, and D are shown in panels E, F, G, and H, respectively. Areas in which the linear trends (positive or negative) have confidence levels larger than 90% are shown in green color in panels E, F, G, and H.

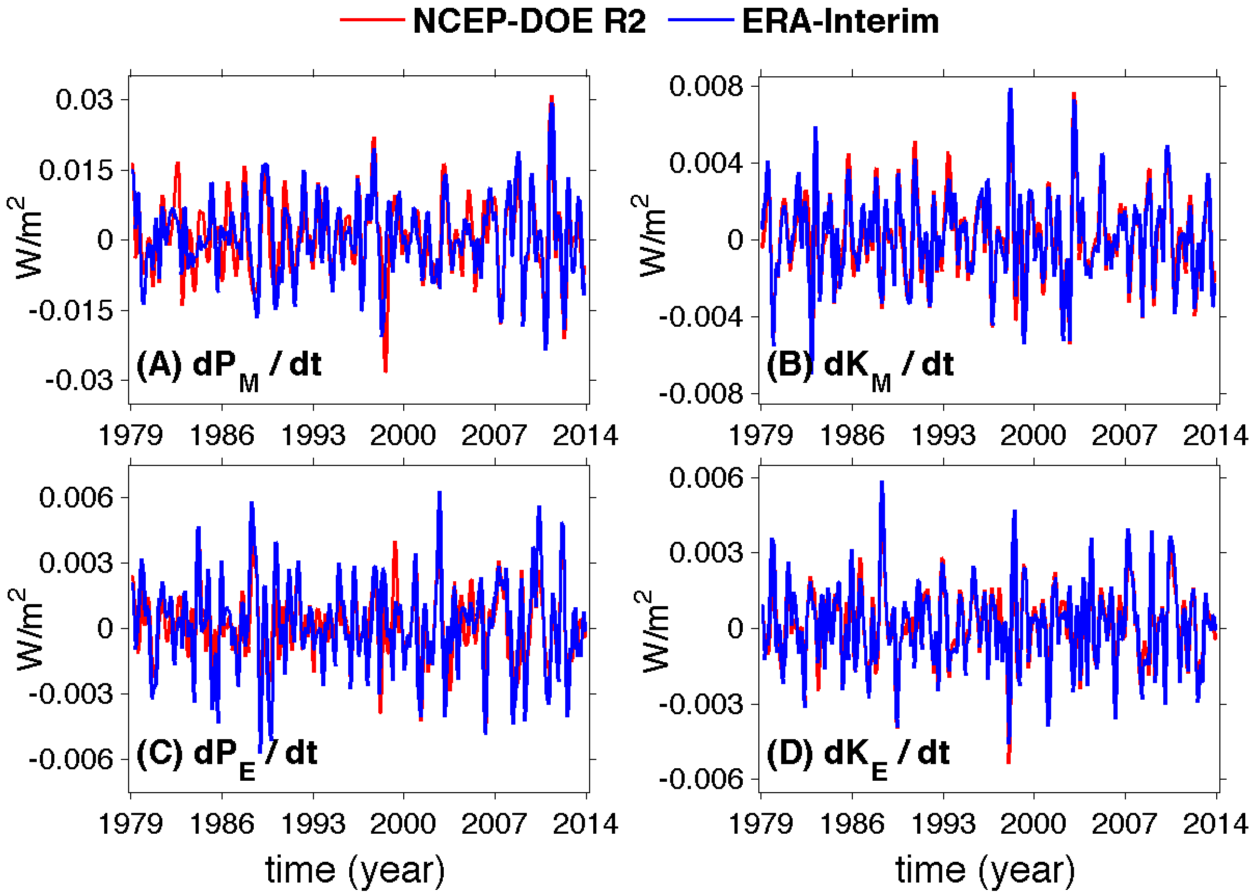

Supplementary Figure 9: Time differentials of energies. The time differentials of energies are based on the time series of energies shown in Fig. 1 in the main text. (A) Time differential of the mean available potential energy dP_M/dt. (B) Time differential of the mean kinetic energy dK_M/dt. (C) Time differential of the eddy available potential energy dP_E/dt. (D) Time differential of the eddy kinetic energy dK_E/dt. The time derivatives of energies are further utilized to compute the generation and dissipation rates of energies, as discussed in the section of Methods.

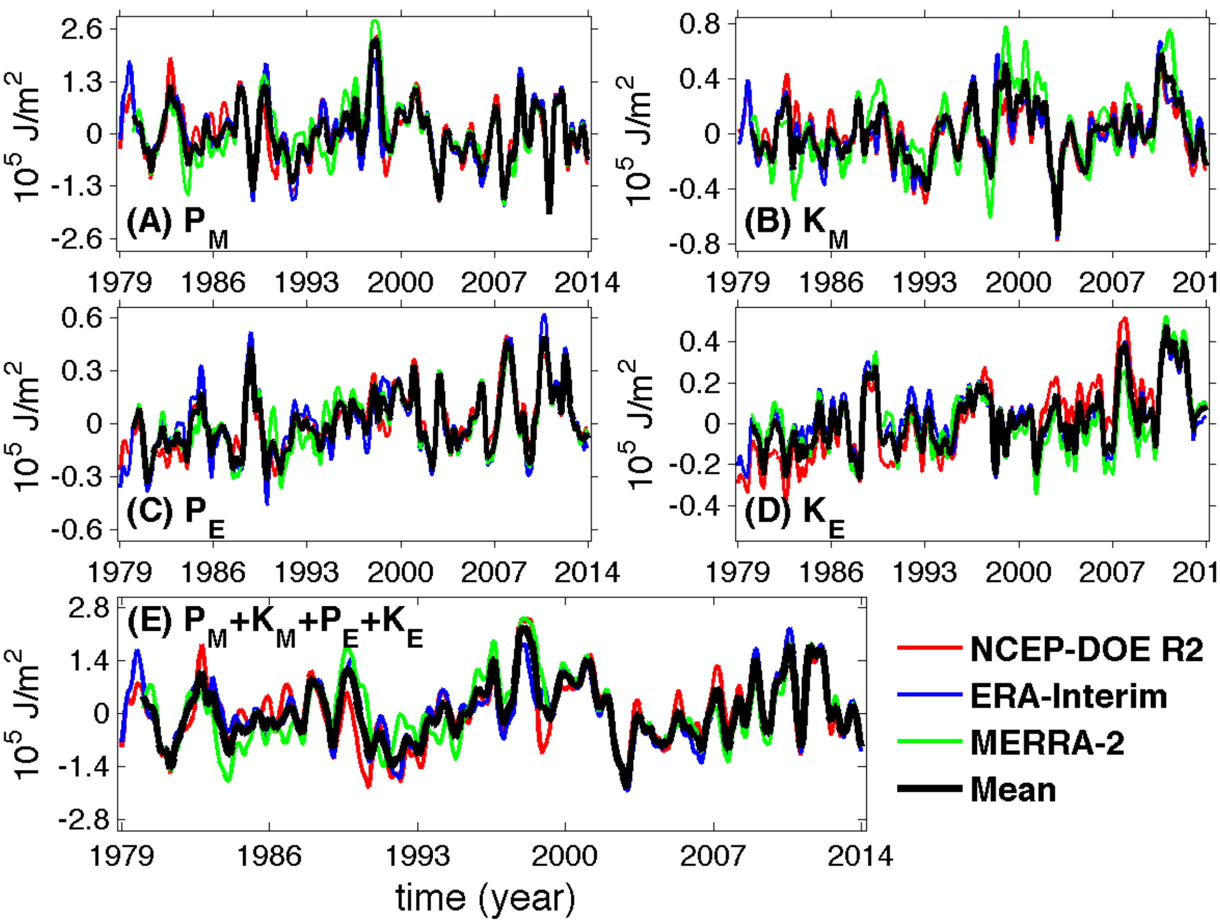

Supplementary Figure 10: Comparison of the global-average atmospheric energies among three data sets. The analyses of NCEP-DOE R2 and ERA-Interim (1979-2013) come from Fig.1 in the main text. The MERRA-2 analyses, which have a different time period (1980-2013), are used to validate the results from the NCEP-DOE R2 and ERA-Interim. In addition, the ensemble mean of the three data sets (NCEP-DOE R2, ERA-Interim, and MERRA-2) are plotted. The linear trends (confidence levels) of the MERRA-2 analyses are -72.7±2008.3 J m^{-2} year^{-1} (<70%), 792.1±812.5 J m^{-2} year^{-1} (83.1%), 584.5±403.7 J m^{-2} year^{-1} (91.0%), 478.0±399.6 J m^{-2} year^{-1} (85.9%), and 1312.9±2645.0 J m^{-2} year^{-1} (<70%) for P_M, K_M, P_E, K_E, and the total mechanical energy (i.e., $P_M + K_M + P_E + K_E$), respectively. Comparing with Table 1 in the main text, we found that the MERRA-2 analyses are basically consistent with the linear trends from the NCEP-DOE R2 and ERA-Interim. Therefore, the MERRA-2 analyses validate the results in the main text (Figure 1 and Table 1). The time series of ensemble mean of the three data sets (1980-2013) generally have the relatively small variances, and hence the corresponding linear trends have higher confidence levels than these of the linear trends from each data set. The linear trends (confidence levels) of the ensemble mean are -320.0±1578.1 J m^{-2} year^{-1} (<70%), 487.5±544.6 J m^{-2} year^{-1} (80.2%), 647.2±411.1 J m^{-2} year^{-1} (93.7%), 734.0±374.0 J m^{-2} year^{-1} (96.6%), and 1158.8±1985.3 J m^{-2} year^{-1} (<70%) for P_M, K_M, P_E, K_E, and the total mechanical energy, respectively.

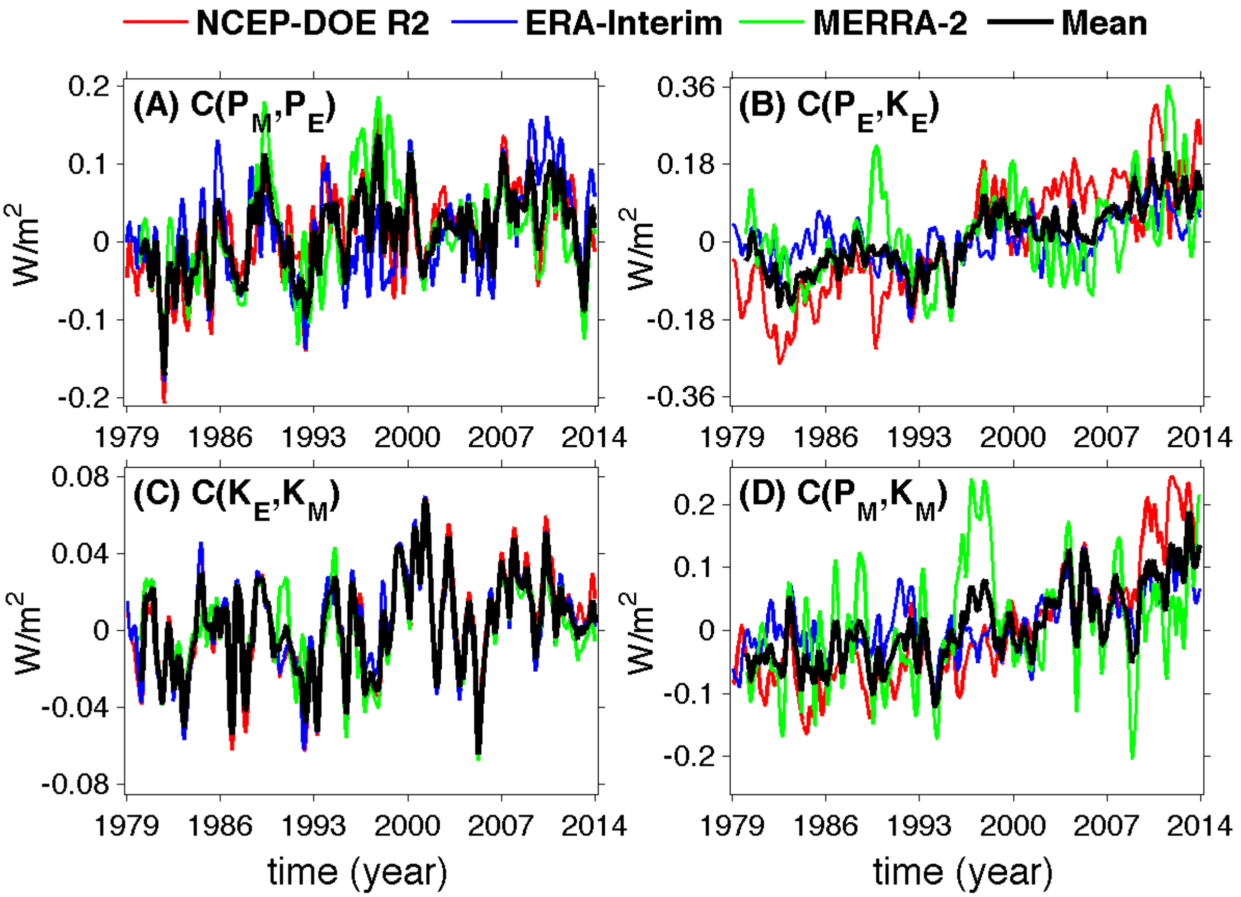

Supplementary Figure 11: Comparison of the global-average conversion rates among three data sets. The analyses of NCEP-DOE R2 and ERA-Interim (1979-2013) come from Fig.2 in the main text. The MERRA-2 analyses, which have a different time period (1980-2013), are used to validate the results from the NCEP-DOE R2 and ERA-Interim. In addition, the ensemble mean of the three data sets (NCEP-DOE R2, ERA-Interim, and MERRA-2) are plotted. The linear trends (confidence levels) of the MERRA-2 analyses are $(1.5\pm1.3)\times10^{-3}$ W m^{-2} year^{-1} (87.3%), $(4.0\pm2.7)\times10^{-3}$ W m^{-2} year^{-1} (92.0%), $(0.5\pm0.5)\times10^{-3}$ W m^{-2} year^{-1} (82.1%), and $(3.1\pm1.9)\times10^{-3}$ W m^{-2} year^{-1} (94.9%) for $C(P_M, P_E)$, $C(P_E, K_E)$, $C(K_E, K_M)$, and $C(P_M, K_M)$, respectively. Comparing with Table 1 in the main text, we found that the MERRA-2 analyses are basically consistent with the linear trends from the NCEP-DOE R2 and ERA-Interim. Therefore, the MERRA-2 analyses validate the results in the main text (Figure 2 and Table 1). The time series of ensemble mean of the three data sets (1980-2013) generally have the relatively small variances, and hence the corresponding linear trends have higher confidence levels than these of the linear trends from each data set. The linear trends (confidence levels) of the ensemble mean are $(2.3\pm1.1)\times10^{-3}$ W m^{-2} year^{-1} (97.1%), $(6.3\pm2.5)\times10^{-3}$ W m^{-2} year^{-1} (96.5%), $(0.8\pm0.5)\times10^{-3}$ W m^{-2} year^{-1} (92.1%), and $(4.6\pm1.7)\times10^{-3}$ W m^{-2} year^{-1} (99.3%) for $C(P_M, P_E)$, $C(P_E, K_E)$, $C(K_E, K_M)$, and $C(P_M, K_M)$, respectively.

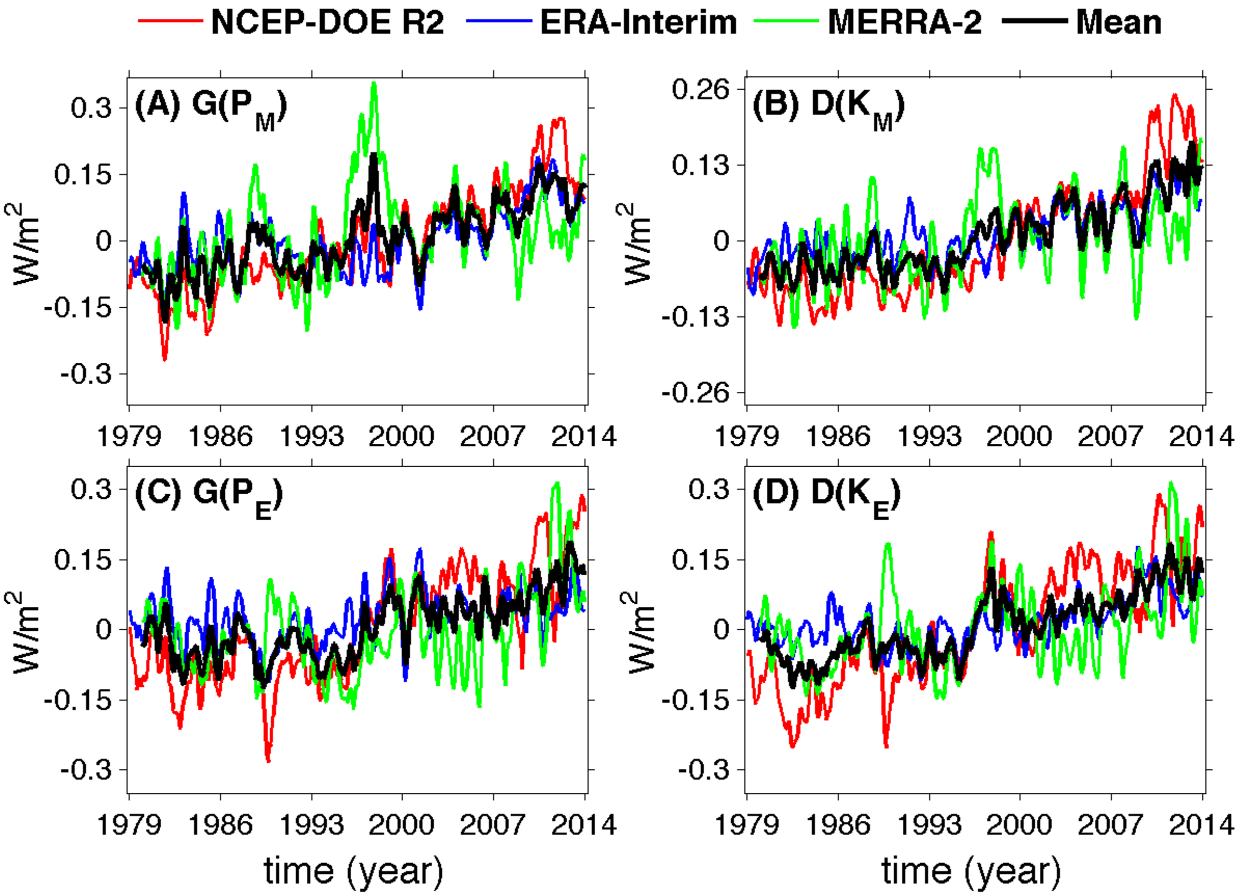

Supplementary Figure 12: Comparison of the global-average generation and dissipation rates among three data sets. The analyses of NCEP-DOE R2 and ERA-Interim (1979-2013) come from Fig.3 in the main text. The MERRA-2 analyses, which have a different time period (1980-2013), are used to validate the results from the NCEP-DOE R2 and ERA-Interim. In addition, the ensemble mean of the three data sets (NCEP-DOE R2, ERA-Interim, and MERRA-2) are plotted. The linear trends (confidence levels) of the MERRA-2 analyses are $(5.8\pm2.1)\times10^{-3}$ W m^{-2} year^{-1} (99.1%), $(5.1\pm1.8)\times10^{-3}$ W m^{-2} year^{-1} (99.4%), $(5.0\pm1.9)\times10^{-3}$ W m^{-2} year^{-1} (99.1%), and $(5.7\pm2.3)\times10^{-3}$ W m^{-2} year^{-1} (96.3%) for $G(P_M)$, $D(K_M)$, $G(P_E)$, and $D(K_E)$, respectively. Comparing with Table 1 in the main text, we found that the MERRA-2 analyses are basically consistent with the linear trends from the NCEP-DOE R2 and ERA-Interim. Therefore, the MERRA-2 analyses validate the results in the main text (Figure 3 and Table 1). The time series of ensemble mean of the three data sets (1980-2013) generally have the relatively small variances, and hence the corresponding linear trends have higher confidence levels than these of the linear trends from each data set. The ensemble mean of the three data sets (1980-2013) generally has smaller variances and higher confidence levels of the linear trends than these of each data set. The linear trends (confidence levels) of the ensemble mean are $(6.1\pm2.3)\times10^{-3}$ W m^{-2} year^{-1} (99.2%), $(5.1\pm1.8)\times10^{-3}$ W m^{-2} year^{-1} (99.4%), $(5.0\pm1.9)\times10^{-3}$ W m^{-2} year^{-1} (99.1%), and $(5.9\pm2.4)\times10^{-3}$ W m^{-2} year^{-1} (96.3%) for $G(P_M)$, $D(K_M)$, $G(P_E)$, and $D(K_E)$, respectively.

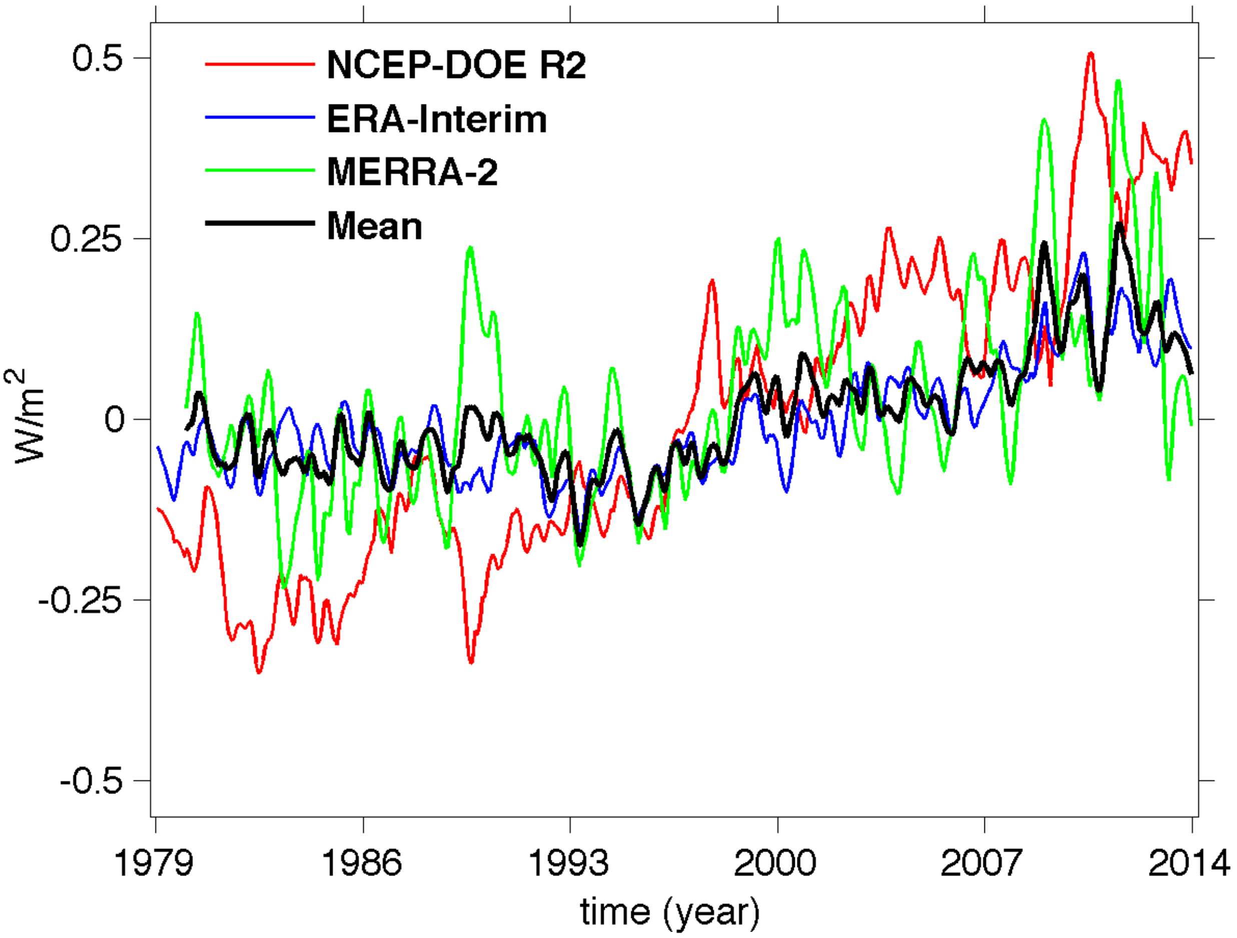

Supplementary Figure 13: Comparison of $D(K_M)+D(K_E)$ among three data sets. The total dissipation rates of the mean and eddy kinetic energies ($D(K_M)+D(K_E)$) are computed from the three data sets (NCEP-DOE R2, ERA-Interim, and MERRA-2). In addition, the ensemble mean of the three data sets are plotted. The linear trend (confidence levels) of the MERRA-2 analyses is $(7.3\pm3.7)\times10^{-3}$ W m^{-2} year^{-1} (94.6%). Comparing with the discussions in the main text, we found that the MERRA-2 analyses are basically consistent with the linear trends from the NCEP-DOE R2 and ERA-Interim. Therefore, the MERRA-2 analyses validate the results in the main text. The time series of ensemble mean of the three data sets (1980-2013) generally have the relatively small variances, and hence the corresponding linear trends have higher confidence levels than these of the linear trends from each data set. The linear trend (confidence levels) of the ensemble mean is $(6.2\pm2.1)\times10^{-3}$ W m^{-2} year^{-1} (96.7%).
